# A Systematic Review of Preclinical Studies Investigating the Effects of Pharmacological Agents on Learning and Memory in Prolonged Aluminum-Exposure-Induced Neurotoxicity

**DOI:** 10.3390/brainsci15080849

**Published:** 2025-08-08

**Authors:** Mahnoor Hayat, Noor Ul Huda Khola, Touqeer Ahmed

**Affiliations:** Neurobiology Laboratory, Department of Biomedicine, Atta-ur-Rahman School of Applied Biosciences, National University of Sciences and Technology, Islamabad 44000, Pakistan

**Keywords:** aluminum neurotoxicity, learning and memory, animal models, systematic review

## Abstract

**Background:** Aluminum accumulation in the brain causes cognitive deficits. No comprehensive synthesis of pharmacological treatments against aluminum neurotoxicity has been conducted, which led us to systematically review the effects of various pharmacological agents against aluminum-induced neurotoxicity, primarily addressing learning and memory after chronic aluminum exposure (≥2 months) in rodent models. **Methods:** A literature search was performed in PubMed, Google Scholar, Science Direct, and Scopus for studies published between 2000 and 2023. A total of 45 studies were selected according to the inclusion criteria. Primary outcomes focused on assessing learning and memory, with 39 different pharmacological agents evaluated explicitly for their effects against aluminum-induced neurotoxicity. Meta-analysis and subgroup analysis were performed to evaluate cognitive improvement in the Morris water maze (MWM) for learning and memory, and oxidative stress parameters were evaluated through superoxide dismutase (SOD) and catalase (CAT) in aluminum-induced neurotoxicity models. **Results:** According to the systematic analysis, most treatments significantly improve learning and memory, except for insulin and melatonin. According to the MWM analysis, Memantine, *Hypericum perforatum* extract, *Bennincasa hespidia*, and, based on the biochemical analysis, Chrysin showed better results. The meta-analysis (random effects) revealed reduced escape latency (SMD = 0.97, 95% CI: 0.74 to 1.19) and increased SOD (SMD = −0.54, 95% CI: −0.79 to −0.29) and CAT levels (SMD = −0.50, 95% CI: −0.73 to −0.27) in treated groups versus aluminum. Egger’s regression tests showed no strong evidence of publication bias. **Conclusions:** This study effectively synthesized preclinical evidence, identifying promising pharmacological agents for mitigating aluminum-induced cognitive deficits. These findings offer a scientific basis for future experimental studies and therapeutic development targeting aluminum neurotoxicity.

## 1. Introduction

Aluminum makes up around 8% of the Earth’s crust and is the third most prevalent element after oxygen and silicon [1,2]. The World Health Organization has determined a tolerable daily aluminum consumption of 1 mg per kg of body weight. Despite this range, human exposure to aluminum exceeds the threshold due to its prevalence in daily life [3]. Aluminum is commonly used in processed and packaged foods [4], pharmaceutical products, such as vaccine adjuvants, and antacids, as well as in food additives, skin care products, cosmetics, and kitchenware [5]. Moreover, it can be present either as a natural component or as a contaminant in several foods, like infant formula, milk, juices, tea, wine, vegetables, and seafood [6]. Drinking water is another common source, where aluminum may be introduced naturally from soil and rock erosion or through water treatment processes [3,7,8,9]. Occupational exposure is also a significant concern, particularly in mining, smelting, and welding industries, where workers are regularly exposed to high levels of aluminum particles [10,11].

In various regions of the world, cases of aluminum toxicity have been documented, raising concerns because of its potential to negatively impact several organ systems, making it a significant global public health concern [12]. Aluminum enters the body through different routes, such as the skin, gastrointestinal and respiratory tracts [11]. Once it enters the body, its bioavailability makes it toxic [13,14]. It accumulates in the liver, kidneys, bones, spleen, blood, testis, heart, muscles, and brain. The brain is most vulnerable to aluminum neurotoxicity. Studies report that the half-life of aluminum in the human brain is almost seven years. Once entered, it has all these years to show its toxic effects [14]. Aluminum assays of blood, urine, hair, nails, and sweat can determine the degree of aluminum poisoning [12].

The effects of aluminum poisoning are complex and multifaceted, including disruptions in enzyme activity, nucleic acid function, cell membrane permeability, and protein synthesis. It impairs DNA repair mechanisms, alters DNA organization stability, and inhibits protein phosphatase 2A (PP2A) activity, leading to hyperphosphorylation in neuronal proteins, like tau and neurofilament proteins [15]. Aluminum crosses the blood–brain barrier (BBB), increases oxidative stress and lipid peroxidation [16,17], and generates reactive oxygen species (ROS) [18]. It disrupts cellular iron homeostasis and activates inflammatory and apoptotic pathways (NF-kB, p53, and JNK), contributing to neuronal death [19]. Additionally, it increases pro-inflammatory cytokines, alters cholinergic neurotransmission, and promotes amyloid-β (Aβ) accumulation and tau hyperphosphorylation, leading to the development of neurodegenerative diseases [14]. Higher aluminum levels in the brain are associated with Alzheimer’s disease (AD), encephalopathy, and Parkinson’s disease (PD) [20,21].

While aluminum’s impact on human health is established, preclinical studies in animal models have provided the primary basis for understanding its toxicological mechanisms. Rodent studies have shown that aluminum exposure leads to significant disruptions in various physiological systems. This systematic review and meta-analysis focus on the evidence from preclinical studies assessing the impact of aluminum in rats and mice. Aluminum exposure causes severe neurotoxicity in rats and mice by accumulating in brain regions, such as the hippocampus and the cortex, the areas engaged explicitly in learning and memory. It disrupts their structure and function [22], resulting in cognitive decline [23,24] and memory impairments [25,26,27,28]. It also alters key signaling molecules and pathways, including BDNF, cAMP, CREB, MAPK, ERK1/2, and CaMKII [29,30], c-jun, NGF, NT-3, and PKC [26,31]. These changes lead to oxidative damage, neuroinflammation, dendritic spine loss, DNA fragmentation, and amyloid plaque formation, ultimately resulting in cognitive decline and neurodegeneration [27,29]. Aluminum also causes significant histological changes, such as neurofibrillary degeneration, neuronal loss, and altered synaptic ultrastructure [25,31,32,33]. Even after developmental exposure, neurogenesis is impaired due to reduced neural stem cell self-renewal and increased ER stress-induced apoptosis [32,34]. Increased Bcl-2 expression in brain areas affected by aluminum could indicate a compensatory anti-apoptotic response [35]. The effects of aluminum on the brain are dose- and time-dependent, causing gradual behavioral, biochemical, molecular, and structural damage.

Knowing the harmful effects of aluminum, it is crucial to examine possible options for its treatment. Different pharmacological treatments have been explored to investigate their impact on aluminum-induced neurotoxicity. Although several studies have investigated treatment strategies against aluminum-induced neurotoxicity, an extensive synthesis of their findings is still lacking, particularly in determining which therapeutic interventions are most effective in mitigating aluminum-induced cognitive impairments and oxidative stress. Therefore, a systematic review is needed to quantitatively synthesize evidence on aluminum-induced oxidative stress and cognitive impairments and assess the efficacy of various pharmacological treatments against it. The present study conducted a comprehensive literature search of in vivo experiments on aluminum-induced neurotoxicity, focusing on learning and memory deficits, oxidative stress markers (e.g., SOD, CAT), and a variety of therapeutic interventions ranging from natural compounds, like Curcumin [36], to synthetic agents such as Naproxen [37] and Memantine [38]. This review provides a valuable approach to identifying consistent patterns from existing evidence and addressing factors related to aluminum neurotoxicity across different studies. This review aims to analyze the efficacy of possible pharmacological treatments and their mechanism of action and guide future research for developing effective treatments against aluminum-induced cognitive impairments.

## 2. Methods

This systematic review was conducted to enlist possible treatments for improving learning and memory affected by aluminum exposure for ≥2 months. After aluminum and treatment exposure, behavioral, molecular, biochemical, and histological patterns were analyzed. Therefore, only studies that include the effects of pharmacological agents on learning and memory in aluminum-induced neurotoxicity were evaluated, as the primary purpose was to find agents that could improve learning and memory in aluminum-induced cognitive impairments. This review was executed as per the guidelines of the Preferred Reporting Items for Systematic Reviews and Meta-Analysis (PRISMA) [39].

### 2.1. Sources of Information and Search Strategies

This systematic review was carried out by searching the following electronic databases: Science Direct, PubMed, and Scopus. Google Scholar was utilized as a secondary search tool. A search was conducted for treatments addressing aluminum-induced neurotoxicity, with a focus on learning and memory outcomes. The search strategy for study selection emphasized the convergence of Medical Subject Headings (MeSH) with the following key terms: aluminum, neurotoxicity, brain OR nervous system, pharmacological treatment OR treatment, and learning and memory. The electronic search was conducted periodically from 2000 to 31 August 2023 in Science Direct, from 2000 to 30 August 2023 in Scopus and PubMed, and from 2000 to 3 September 2023 in Google Scholar.

The search strategy was designed according to the characteristics of each database. For Science Direct, the subject areas included for selection criteria were Neuroscience (189), Pharmacology, Toxicology, and Pharmaceutical Science (218), and, for Google Scholar, the search term used was aluminum neurotoxicity pharmacological OR treatment OR learning OR and OR memory, and, in a modified search, “title of the studies” was selected. We searched up to September 2023, and all searched files were downloaded once, compiled, and then screened manually by individual reviewers.

### 2.2. Selection of Studies and Data Collection

In the initial step of screening and selecting the studies, duplicates were removed from the downloaded Excel sheets using the duplicate remover and then checked independently by the reviewers; no automation tools were used. During the initial screening, 653 records were found through all database searches, 107 duplicates among different database searches were removed, and 338 studies were excluded by reading the titles of the studies and abstracts according to predetermined inclusion–exclusion criteria. All of the studies that did not assess treatment for aluminum neurotoxicity or did not assess learning and memory outcomes were excluded. The primary objective of this systematic review was to identify potential treatments for learning and memory affected by aluminum neurotoxicity. Those studies in which any combination of chemicals as a neurotoxin, i.e., d-galactose, arsenic, selenium, etc., were administered alongside aluminum were also excluded. Finally, those studies were selected in which aluminum was administered for two months or more without any chemical combination, followed by a pharmacological intervention to assess its effects on learning and memory using behavioral tests, such as the MWM [40] and the Y maze [41]. Studies involving aluminum exposure longer than two months were selected to enhance the reliability and relevance of the findings to long-term neurobehavioral outcomes, as chronic exposure to environmental sources more accurately reflects human environmental and occupational exposure scenarios. Additionally, two months of sub-chronic exposure to low doses of aluminum from feed and water, which reflects human dietary aluminum intake, has been demonstrated to reach a threshold sufficient for promoting memory impairment and neurotoxicity in rats [42].

**Figure 1:**
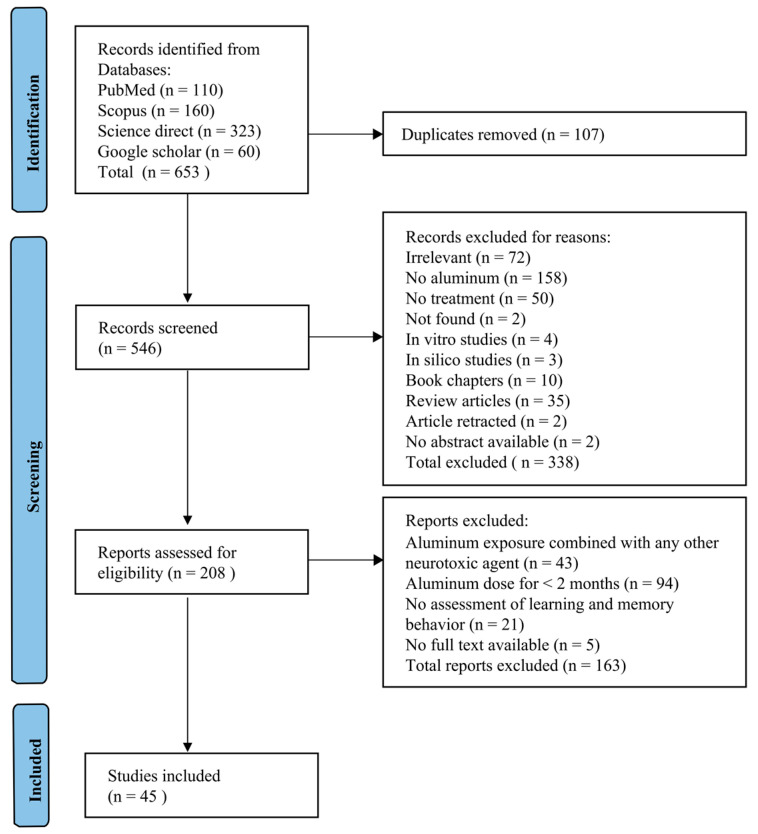
PRISMA flow diagram of study selection.

The detailed results of the exclusion and inclusion parameters following the PICO parameters [43,44] are shown in the PRISMA diagram in Figure 1.

### 2.3. Eligibility Criteria

Studies were eligible for inclusion if they were original research articles that involved aluminum-induced neurotoxicity, assessed learning and memory outcomes, and evaluated the therapeutic effects of pharmacological interventions compared to an aluminum group. The details are mentioned in Table 1.

### 2.4. Data Extraction

For each study included in the systematic review, the following information was extracted: study characteristics (title, author(s), and publication year), experimental subjects (sex, species, strain (if available), weight, age, and number of subjects), aluminum and pharmacological agent exposure parameters (form, administered dose, duration, and route), and experimental results (behavioral, biochemical, histological, and genetic expression analysis). The characteristics of the included studies were summarized in Table 2, and experimental results of each study are detailed in Table 3.

Among the numerous behavioral tests reported in the included studies, the MWM was the most used behavioral test, reported in 29 studies. Escape latency was the only standard parameter evaluated across 28 studies, as at least one shared parameter is essential for meaningful comparison. Sixteen different behaviors were analyzed in selected studies; however, not all tests provided sufficient evidence for a robust statistical comparison due to their limited representation. The list of behaviors reported in the shortlisted studies is mentioned in Table 4. Among biochemical parameters, SOD and CAT are essential anti-oxidant enzymes that are well known for their roles in neutralizing ROS, making them reliable indicators of oxidative stress. SOD and CAT activity was assessed in 21 and 18 studies, respectively. Other oxidative stress markers reported were LPO, GPx, etc. However, they were reported in fewer studies, providing insufficient evidence for quantitative synthesis or comparison across a wider dataset. Therefore, for the meta-analysis, those studies were selected that reported escape latency of MWM, SOD, and CAT outcomes. The number of animals per group (i.e., aluminum-treated group and aluminum + treatment group), mean results of these groups, and their standard deviation were extracted to make a forest plot of these studies. As many studies reported outcomes in graphical form, we used WebPlotDigitizer v4.8 to accurately extract numerical values from graphs, which were then used for forest plot generation. WebPlotDigitizer is an online computer-vision-assisted program that assists in extracting numerical data from images of various data visualizations. In cases where different treatment doses were given, the high-dose result was selected. Most of the time, the highest dose used has shown better results. If data were collected at different time points during the study, the data at the end of the treatment were selected.

### 2.5. Methodological Quality, Transparency, and Reporting Standards of Studies

Initially, studies were screened without limiting species; however, after applying the inclusion criteria and narrowing the selection based on study relevance, only animal studies involving rats and mice met the requirements for inclusion in this systematic review. Studies that did not meet specific inclusion criteria, such as those lacking a treatment intervention or relevant behavioral assessments, were excluded. Therefore, quality evaluation criteria were made keeping in view the animal experimental systems designed by [45] and according to the CAMARADES study quality checklist [46,47]. There were 8 evaluation categories; if the study fulfilled the criteria in one category, one point was assigned. The highest possible score was 8. The methodological quality of studies was evaluated using the following criteria: (1) is the publication peer-reviewed? (2) were animals randomly allocated in groups? (3) is the housing temperature reported? (4) is the sex of the animals reported? (5) is the strain of the animals reported? (6) avoidance of anaesthetic agents with neuroprotective properties (ketamine, sodium pentobarbital, barbitone, thiopental sodium, halothane), (7) is the sample size mentioned? (8) declaration of potential conflicts of interest. Each study was assessed against 8 criteria, outlined in Table 5. A cumulative score was used to classify methodological quality and reporting standards as follows: good (6–8), fair (3–5), and poor (0–2). Scoring was based on the number of criteria fulfilled. In total, 38 studies were categorized as good quality, 7 as fair quality, and none as poor quality based on the scoring criteria.

Furthermore, some questions were assessed regarding studies’ Transparency and Reporting Standards. This category evaluated the clarity and completeness of studies and whether all of the information necessary to reproduce the experiment was included in the study. For this reason, clarity in a report is essential to allow a study to be repeated and to facilitate comparison. The key elements addressed were (1) aluminum dose mentioned, (2) treatment agent dose mentioned, (3) duration of treatment mentioned, (4) model validation, (5) toxicity of agent evaluated, (6) compliance with animal welfare regulations or animal ethical approval, (7) euthanasia protocol mentioned, (8) demographic data (sex, strain, weight, age of animals) (1 point for each trait, for a total of 4), (9) factors assessed (histology, biochemical, molecular assessment), with 1 point for each, and (10) number of behaviors assessed (if ≥3 = 1, if ≤2 = 0). There were 10 evaluation categories. If the study fulfilled the criterion in one category, a score of one point was given. The maximum possible score was 15 (Table 6).

### 2.6. Statistical Data Analysis

The amount of the difference or the intensity of the difference between variables was measured based on effect size [48]. The standardized mean difference (SMD) was used to measure effect size because it allows for comparisons between studies that employ different scales; SMD was used to assess outcomes (e.g., escape latency of MWM, SOD, and CAT levels) between aluminum + treatment and aluminum-exposed groups. This approach allows us to measure the degree of treatment effects in a standardized way across studies using different scales. Review Manager version 5.4 software was used to analyze the data. Group means, standard deviations, and sample sizes taken from the included studies were used to calculate SMDs in Review Manager. To assess continuous variables between the aluminum + treatment agents’ group and the aluminum-treated group, SMDs with a 95% confidence interval (CI) were used. We used 95% CI to indicate the precision and reliability of the estimated effect sizes. A 95% CI reflects the range in which the true effect is likely to fall within 95% certainty. Using the SMD and 95% CI, the therapeutic value of the treatment agent was calculated. According to the *Cochrane Handbook*, the SMD used in Review Manager applies the correction for small sample sizes and corresponds to Hedges’ g [49,50], which is an adjusted version of Cohen’s d. This adjustment ensures more accurate effect size estimation when included studies have small sample sizes [51,52]. A random effects model was chosen [53,54], given the expected heterogeneity in study design, animal strains, and dosing regimens. I_2_ was used to measure heterogeneity across studies. If I_2_ is low (less than 25%), most of the variation is likely due to chance. A value between 30% and 60% indicates moderate heterogeneity. But, if it is high (more than 50%), it suggests real differences between studies. A value of 50% to 90% indicates substantial heterogeneity, and 75 to 100% indicates significant heterogeneity. The I_2_ statistic is used to evaluate the extent of the inevitable heterogeneity among studies [55,56]. A significance level of 0.05 was used. Subgroup analysis was applied to assess the degree to which different treatment agents enhanced memory.

Using Jamovi software v2.6.26, publication bias was assessed using Egger’s regression test, a statistical method used to identify publication bias in meta-analyses [57]. It evaluates a funnel plot’s asymmetry, which is a scatter plot of effect sizes vs. standard errors. A substantial Egger’s test result raises the possibility of publication bias, which could cause the meta-analysis to overestimate treatment effects. Studentized residuals and Cook’s distances were used to examine whether studies may be outliers and/or influential in the context of the model [58]. Studies with a Cook’s distance larger than the median plus six times the interquartile range of the Cook’s distances were considered to be influential.

## 3. Results

### 3.1. Study Inclusion

A total of 653 studies were identified initially (323 from Science Direct, 160 from Scopus, 110 from PubMed, and 60 from Google Scholar). When titles and abstracts were screened, 208 studies remained. Furthermore, 43 studies were eliminated as they had used a combination with aluminum, 94 studies were eliminated because they had less than 2 months of aluminum intervention, and 21 studies had not reported any learning and memory behavior. Five studies with no full text available were also excluded. Finally, 45 animal studies were selected and included in the systematic review.

In the selected studies, two kinds of animals, rats (33 studies) and mice (12 studies), were used. Among them, 32 studies mentioned oral (p.o.), 2 subcutaneous (s.c.), and 11 intraperitoneal (i.p.) routes of aluminum administration. Treatment agents were administered via oral (36 studies) and i.p. (9 studies) routes. A total of 21 studies used two or more doses of treatment in different groups. Out of the total of 45 studies, 36 used male animals (28 rats, 8 mice), 5 studies used female animals (2 rats, 3 mice), and 3 studies used both male and female (among them, 2 studies included rats and 1 study included mice of either sex). One study did not mention the sex of the animals [59], and one study used a transgenic mouse model [60].

### 3.2. Study Characteristics

Cognitive function impairment is one of the symptoms of aluminum neurotoxicity; thus, the pharmacological agent should possess qualities to enhance cognitive function. Animal models were commonly used in many studies to observe metal neurotoxicity. In these retrieved studies on aluminum neurotoxicity, aluminum in different forms was used, i.e., two studies reported aluminum gluconate, one study reported aluminum lactate [60], and one study reported AlCl_3_ hexahydrate [37]. The rest of the 41 studies reported aluminum chloride (AlCl_3_) for aluminum neurotoxicity induction. Different aluminum administration routes were used, such as subcutaneous and intraperitoneal injection, oral gavage, or in drinking water. All of these characteristics, including the chemical form, dose, duration, and route of administration of aluminum, as well as the treatment agent, are mentioned in Table 2. A pharmacological agent is a substance or drug that is used to identify, treat, or prevent diseases or medical conditions. To provide a clearer understanding of the therapeutic potential of various agents against aluminum-induced neurotoxicity, the included agents are categorized based on the type of pharmacological agent used. Synthetic FDA-approved drugs, phytochemicals and plant extracts, herbo-mineral compounds, nutraceuticals and anti-oxidants, hormonal or peptide agents are among the therapeutic agents evaluated in the included studies. Synthetic FDA-approved drugs include Rivastigmine, Memantine, Naproxen, Citicoline, Azilsartan, and Perindopril. Phytochemicals and plant extracts include curcumin, hesperidin, naringin, Ginsenoside Rb1, Icariin, Chrysin, *Echinacea purpurea*, *Centella asiatica*, *Cassia tora*, *Hypericum perforatum*, pomegranate juice, *Pistacia lentiscus*, betulin, *Fraxinus angustifolia*, apple polyphenol extract, etc. Herbo-mineral compounds/polyherbal formulations include Rasa Sindoor and Manasamitra vatakam. Nutraceuticals and anti-oxidants include melatonin, EGCG (free and nano), soy isoflavones, and tannoids of *Emblica officinalis*. Hormonal or peptide agents include insulin. Prostaglandin analogs include Misoprostol and Beraprost sodium. Most agents exert their effects through anti-oxidant and anti-inflammatory mechanisms, which are central to mitigating aluminum-induced neurotoxicity. Specifically, agents like curcumin, hesperidin, melatonin, naringin, *Centella asiatica*, and *Ginkgo biloba* have well-documented anti-oxidant properties that reduce lipid peroxidation, enhance endogenous anti-oxidant enzymes (SOD, CAT, GPx), and protect neuronal structures. Others, like Rivastigmine and Memantine, exert neurotransmission-modulating effects, improving cognitive functions through cholinergic or glutamatergic pathways. Several plant-based extracts (e.g., *Benincasa hispida*, *Cassia tora*, pomegranate juice) and polyphenols (e.g., apple polyphenol extract, betulin) show multi-target effects by reducing oxidative stress and suppressing neuroinflammation. Agents like Azilsartan, Perindopril, and Beraprost sodium, though primarily vasoactive drugs, also demonstrate neuroprotective roles by improving cerebral blood flow and reducing oxidative damage. *Hypericum perforatum* is a plant extract rich in phytochemicals, such as hypericin and flavonoids, and known for its anti-oxidant and anti-inflammatory properties, which contribute to improved memory and reduced oxidative stress in treated animals. *Benincasa hispida* (Ash gourd), a medicinal plant widely used in traditional Ayurvedic formulations, contains bioactive compounds like flavonoids and sterols that exert strong anti-oxidant and neuroprotective effects. Its administration significantly reversed behavioral and biochemical alterations in aluminum-exposed rodents, supporting its potential role in cognitive enhancement.

**Table 2:** Characteristics of the included studies.

Study	Aluminum, Dose, Duration, and Route	Pharmacological Agent, Dose, Duration, and Route	Combination	Animal Sex, Strain, Age, and Weight	Anaesthesia Dose	Behaviors Accessing Learning and Memory	Properties of Pharmacological Agent
Abdel-Aal et al., 2011 [38]	AlCl_3_ 100 mg/kg 60 days i.p.	Memantine 5, 10, and 20 mg/kg 60 days i.p.	-	Male Wistar rats (90–100 days old) 190 to 230 g	N/M (no dissection)	MWM, PAT, RAM, OFT, RRT	Enhance cognitive functions
Abdel-Aal et al., 2011 [61]	AlCl_3_ 100 mg/kg 60 days i.p.	Rivastigmine 0.5, 1, 1.5, and 2.5 mg/kg 60 days i.p.	-	Male Wistar albino rats (90 days old) 190–240 g	N/M (no dissection)	OFT, MWM, RAM, PAT, RRT	Cholinesterase inhibition
Abdel-Aal et al., 2022 [37]	AlCl_3_ hexahydrate 80 mg/kg 60 days i.p.	Naproxen 20 mg/kg 14 days i.p.	Rivastigmine (1 mg/kg)	Male albino Wistar rats (age N/M) 180–220 g	Thiopental sodium (50 mg/kg)	NOR, PAT, MWM	Cholinergic inhibition Anti-inflammatory Anti-apoptotic
Abdel-Zaher et al., 2017 [62]	AlCl_3_ 100 mg/kg 90 days i.p.	Citicoline 100 mg/kg 90 days i.p.	-	Male albino Wistar rats (age N/M) 180–220 g	N/M	MWM, PAT, RAM	Anti-oxidant
Allagui et al., 2014 [63]	AlCl_3_ 50 mg/kg 120 days Oral gavage	Melatonin 10 mg/kg 120 days i.p.	-	Male Wistar rats Young (60 days) and old (720 days old) Weight N/M	N/M	OFT, RAM, EPM	Anti-oxidant Neuroprotective Cognitive enhancement Enhance neuronal health
Alzahrani et al., 2020 [64]	AlCl_3_ 100 mg/kg 60 days Oral	Azilsartan (3.5 mg and 7 mg/kg), Perindopril (0.5 mg and 1 mg/kg), 60 days Oral gavage	-	Male Wister rats (42–56 days old) 180–220 g	N/M	Y maze	Anti-inflammatory (reduces TNF-α) Reduces amyloidogenic activity Anti-oxidant (reduces lipid peroxidation)
Amjad and Umesalma, 2015 [65]	AlCl_3_ 300 mg/kg 60 days Oral	*Centella asiatica* 500 mg/kg 60 days Oral	-	Either sex Wistar albino rats (age N/M) 120–150 g	Diethyl ether (dose N/M)	HWM, LA by actophotometer, RRT	Anti-oxidant Restore cholinergic neurotransmission
Azib et al., 2019 [66]	AlCl_3_ 100 p.p.m./day 60 days Oral	*Pistacia lentiscus* L. leaves extract 150 and 300 p.p.m./day 60 days Oral	-	Male adult albino mice (age N/M) 18–25 g	N/M	Head dipping, Black and White test, EPM, MWM	Prevents oxidative stress and lipid peroxidation Anti-inflammatory
Azib et al., 2020 [67]	AlCl_3_ 100 mg/kg 60 days Oral	*Fraxinus angustifolia Vahl*. bark extract 150 and 300 mg/kg 60 days Oral	-	Male NMRI mice (age N/M) 26 ± 2 g	N/M	LA, Black and White test, MWM	Anti-oxidant Anti-inflammatory Anti-apoptotic Direct inhibition of Aβ-aggregation
Bhargava et al., 2023 [14]	AlCl_3_ 100 mg/kg 60 days Oral gavage	*Cassia tora* extract 300 mg/kg 60 days Oral gavage	Memantine (20 mg/kg)	Male Wistar rats (age N/M) 200 ± 20 g	Halothane (dose N/M)	MWM	Anti-oxidant Anti-inflammatory Cognitive enhancement Neurotransmitter regulation
Campos et al., 2022 [68]	AlCl_3_ 100 mg/kg 90 days Oral	Chrysin 10, 30, and 100 mg/kg 45 days Oral gavage	-	Male Swiss mice (about 60 days old) 25–30 g	Ketamine and xylazine hydrochloride (dose N/M)	OFT, Chimney Test, Step-Down Avoidance	Anti-oxidant Anti-inflammatory Neuroprotective
Cao et al., 2017 [69]	AlCl_3_ 150 mg/kg 90 days Oral	*Hypericum perforatum* extract 150 and 300 mg/kg 60 days Oral	-	Male Wistar rats (42–49 days old) 185–200 g	Sodium pentobarbital (dose N/M)	OFT, MWM	Anti-inflammatory Anti-oxidative Neuroprotective Learning and memory enhancing
Cheng et al., 2014 [70]	AlCl_3_ 171.8 mg/kg 70 days Oral	Apple (Ralls) polyphenol extract 200 mg/kg 70 days Oral	-	Male Wistar rats (49 days old) 160–180 g	N/M	SDIA, MWM	Anti-oxidant Metal-chelating Anti-apoptotic
Dibacto et al., 2022 [71]	AlCl_3_ 75 mg/kg 60 days Esophageal gavage	*Xylopia parviflora* 150 and 300 mg/kg 60 days Esophageal gavage	Donepezil (5 mg/kg) and curcumin (100 mg/kg)	Female Wistar rats (456 days old) 200–220 g	N/M	MWM, OFT	Anti-oxidant Cholinesterase inhibition
Firdaus et al., 2022 [21]	AlCl_3_ 100 mg/kg 60 days Oral	*Centella asiatica* 150, 300 mg/kg 60 days Oral	-	Male Charles Foster strain rats (age N/M) 200–260 g	N/M	Y maze, OFT	AchE inhibition Cognitive enhancement Cellular protection
Gadouche et al., 2018 [72]	AlCl_3_ 500 mg/kg 84 days Oral	Pomegranate juice 500 mg/kg 90 days Oral	-	Female Swiss albino mice (428 days old) 18.5 ± 1.98 g	N/M	LA, FST, EPM, MWM	Anti-inflammatory Anti-oxidant Anti-apoptotic
García et al., 2009 [60]	Aluminum lactate 1 mg of Al/g of diet 183 days Oral	Melatonin 10 mg/kg 183 days Oral	-	Female transgenic (Tg2576) and wild-type mice (152 days old) Weight N/M	Ketamine (80 mg/kg) and xylazine (10 mg/kg)	OFT, MWM	No significant protective effect
Gong et al., 2005 [73]	AlCl_3_ 500 mg/kg (60 days, i.g.) + 1600 p.p.m. (up to 152 days, in drinking water) Up to 183 days Oral	*Ginkgo biloba* leaf extract 50, 100, and 200 mg/kg 60 days i.g.	-	Male Wistar rats (56–84 days old) 200–250 g	35% chloral hydrate (dose N/M)	MWM	Anti-oxidant Neuroprotective Anti-apoptotic
Gothwal et al., 2019 [74]	AlCl_3_ 100 mg/kg 60 days i.p.	Rivastigmine (in 3 delivery systems: pure RIV, PAMAM- RIV, Lf-RIV) 2 mg/kg 14 days i.p.	-	Either sex Swiss albino mice (20–25 g) (age N/M)	N/M	ORM	Neuroprotective Cognitive enhancer Cholinergic-supportive as it inhibits AChE
Guo et al., 2016 [75]	Aluminum gluconate 200 mg/kg 100 days i.g.	Misoprostol 30, 60, and 120 μg/kg 100 days i.g	-	Sprague Dawley male rats (age N/M) 200–250 g	N/M	MWM	Anti-inflammatory Anti-oxidant Anti-apoptotic effects (by modulating the PGES-PGE2-EPs signaling pathway)
Justin Thenmozhi et al., 2017 [76]	AlCl_3_ 100 mg/kg 60 days i.p.	Hesperidin 100 mg/kg 60 days i.g.	-	Male albino Wistar rats (70–84 days old) 200–225 g	N/M	RAM, EPM, PAT	Improve learning and memory Anti-oxidative Anti-apoptotic
Justin-Thenmozhi et al., 2018 [77]	AlCl_3_ 100 mg/kg 60 days i.p.	Hesperidin 100 mg/kg 60 days i.g.	-	Male albino Wistar rats (70–84 days old) 200–225 g	Ketamine chloride (24 mg/kg)	Y maze, NOR, RRT	Anti-inflammatory Anti-oxidative Anti-apoptotic Neuroprotective Learning and memory enhancing
Kakkar and Kaur, 2011 [78]	AlCl_3_ 100 mg/kg 126 days Oral gavage	Curcumin (free curcumin: 50 mg/kg) (solid lipid nanoparticles of curcumin: 1, 12.5, 25, 50 mg/kg) 42 days Oral gavage	Rivastigmine (1.5 mg/kg)	Male Lacca mice (56–84 days old) 15–25 g	N/M	MWM	Improve brain histopathology Improve cognition Anti-oxidant
Kumar et al., 2019 [79]	AlCl_3_ 100 mg/kg 60 days Oral	Artesunate 28 mg/kg + Rivastigmine 1 mg/kg 60 days Oral	Memantine (20 mg/kg)	Male Wistar albino rats (age N/M) 150–250 g	N/M	PAT	Memory enhancing Anti-inflammatory Synergistic
Li et al., 2018 [80]	AlCl_3_ 50 mg/kg 60 days Subcutaneous injection	Isorhynchophylline 20 and 40 mg/kg 56 days i.g.	Donepezil (5 mg/kg)	Male Balb-c mice (121 days old) 25–30 g	N/M	RAM	Anti-oxidant (suppress NF-κB signaling pathway) Enhance learning and memory
Liu et al., 2010 [81]	AlCl_3_ 100 mg/kg 60 days i.p.	Soy isoflavones 60 mg/kg 60 days i.p.	-	Kunming male mice (age N/M) 20 g	N/M	PAT	Improve learning and memory
Luo et al., 2007 [82]	AlCl_3_ 1600 p.p.m. 243 days Oral	Icariin 60 and 120 mg/kg 91 days Oral gavage	-	Male Wistar rats (age N/M) 400–500 g	N/M	MWM	Anti-oxidant effects Decreased Aβ_1–40_ levels
Mohamed et al., 2023 [83]	AlCl_3_ 175 mg/kg 60 days Oral	*Echinacea purpurea* extract 250 mg/kg 60 days Oral	Rivastigmine (0.3 mg/kg)	Male Wistar rats (age N/M) 150–170 g	Thiopental sodium (50 mg/kg)	Y maze, FST, NOR	Anti-oxidant Learning and memory enhancing AchE inhibitor
Nampoothiri et al., 2017 [84]	AlCl_3_ 175 mg/kg 60 days Oral gavage	Insulin 0.5 insulin units/kg 60 days i.p.	Glucose (200 mg/kg), Rivastigmine (1 mg/kg)	Male Wistar rats (90 days old) 200–220 g	N/M	MWM	No significant neuroprotective properties
Nehru and Bhalla, 2007 [85]	AlCl_3_ 40 mg/kg 60 days Oral gavage	Centrophenoxine 100 mg/kg 42 days after Al i.p.	-	Female Sprague Dawley rats (age N/M) 160–200 g	N/M	AAT, PAT	Cholinergic and cognitive enhancing Neurotransmitter restorative
Pan et al., 2015 [86]	Aluminum gluconate 200 mg/kg 100 days i.g.	Beraprost sodium 6, 12, and 24 μg/kg 100 days i.g.	-	Male Sprague Dawley rats (56 days old) 200–250 g	4% chloral hydrate (1 mL/100 g)	MWM	Anti-oxidant Anti-inflammatory Neuroprotective
Prabhakar, 2020 [87]	AlCl_3_ 100 mg/kg 60 days i.p.	Naringin 25, 50, and 100 mg/kg 30 days Oral	Donepezil (0.75 mg/kg)	Either sex Wistar rats (age N/M) 180–210 g	N/M	OFT, RAM, RRT, MWM	Anti-oxidant Neuroprotective Cholinergic restoration
Qi-Hai et al., 2006 [88]	AlCl_3_ 50 g/L (30 days, gastrogavage) + 1.6 g/L (60 days, in drinking water) 3 months Oral	*Ginkgo biloba* leaf extract 50, 100, 200 mg/kg 60 days Oral	-	Male Wistar rats (56–84 days old) 200–250 g	35% chloral hydrate (1 mL/kg)	MWM	Anti-oxidant Inhibit AChE expression Neuroprotective
Rapaka et al., 2021 [89]	AlCl_3_ 100 mg/kg 168 days Oral	*Benincasa hispida* 250, 500 mg/kg 112 days Oral gavage	-	Male Sprague-Dawley rats (84 days old) 200–250 g	Ether anaesthesia (dose N/M)	MWM, Y maze	Anti-inflammatory Anti-oxidative Neuroprotective Learning and memory enhancing
Ravi et al., 2018 [20]	AlCl_3_ 300 mg/kg 60 days Oral catheter	*Caesalpinia crista* methanolic extract 100 and 400 mg/kg 60 days Oral catheter	Rivastigmine (1.5 mg/kg)	Male Wistar albino rats (age N/M) 180–200 g	N/M	MWM, LA	AChE inhibition Anti-oxidant Anti-inflammatory
Ravi et al., 2020 [90]	AlCl_3_ 300 mg/kg 60 days Oral catheter	*Cassia tora* extract 100, 400 mg/kg 60 days Oral catheter	Rivastigmine (1.5 mg/kg)	Male Wistar albino rats (age N/M) 180–200 g	Sodium pentobarbitone (100 mg/kg)	MWM, LEA	Anti-amyloid aggregation Memory and cognitive protection Neuroprotective Anti-oxidant Anti-inflammatory Pro-neurotrophic Cholinergic protection Improvement in motor skills
Rijal et al., 2019 [91]	AlCl_3_ 10 mg/kg 60 days i.p.	Phenylpropanoids; para-methoxycinnamic acid and ethyl-p-methoxycinnamate 50 mg and 100 mg/kg 20 days Oral	Rivastigmine (1 mg/kg) and Memantine (20 mg/kg)	Male Wistar rats (121 days old) 250–300g	N/M	OFT, MWM	Anti-oxidant Neuroprotective (AChE modulation, reduces oxidative stress)
Saba et al., 2017 [23]	AlCl_3_ 40 mg/kg 60 days i.g.	Rasa Sindoor 2 g/kg 30 days i.g.	-	C57BL/6J male mice (60 days old) Weight N/M	Urethane (1.5 g/kg)	MWM	Improve cognitive functions and memory Maintain neurotransmitter cycling and GABAergic TCA cycle
Sethi et al., 2009 [36]	AlCl_3_ 50 mg/kg 180 days Oral	Curcumin 30 mg/kg 180 days Oral gavage	-	Male albino Wistar rats Young (121 days old) and old (547 days old) Weight N/M	Ketamine (80 mg/kg) and xylazine (10 mg/kg)	MWM, OFT	Anti-oxidant activity Membrane stabilization PKC regulation Neuronal protection Cognitive enhancement Anxiolytic effects
Shalaby et al., 2023 [92]	AlCl_3_ 50 mg/kg 60 days Subcutaneous injection	Ginsenoside Rb1 70 mg/kg 60 days Oral	-	Male albino mice (121 days old) 25–30 g	5% chloral hydrate (dose N/M)	PAT	Anti-oxidant Anti-apoptotic Aβ and tau inhibition Anti-inflammatory
Singh et al., 2018 [24]	AlCl_3_ 100 mg/kg 60 days Oral gavage	EGCG (Epigallocatechin-gallate) and EGCG-loaded nanoparticles 10 mg/kg 30 days Oral gavage	-	Male Swiss albino Wistar rats (age N/M) 200–250 g	N/M	MWM, OFT, NOR	Anti-amyloidogenic Anti-oxidant Neuroprotective Cholinergic restoration
Thenmozhi et al., 2016 [93]	AlCl_3_ 100 mg/kg 60 days i.p.	Tannoids of *Emblica officinalis* 100 mg/kg 60 days Oral	-	Male albino Wistar rats (70–84 days old) 200–225 g	N/M	PAT, EPM, RAM	Anti-oxidant Anti-apoptotic Prevent tau hyperphosphorylation (by activating the Akt/GSK-3β signaling pathway)
Thirunavukkarasu et al., 2012 [94]	AlCl_3_ 100 mg/kg 90 days Oral	Manasamitra vatakam 100 mg/kg 90 days Oral	-	Male adult Wistar albino rats (age N/M) 200–220 g	N/M	EPM, AAT	Cell-protective Anti-oxidant Anti-inflammatory
Zakrzeska et al., 2023 [59]	AlCl_3_ 200 mg/kg 120 days Oral gavage	Betulin 100 mg/kg 60 days i.g.	Cyclodextrin (100 mg/kg)	Adult Wistar rats (age N/M) 220–250 g	Ether anaesthesia (dose N/M)	MWM, BWT	Anti-oxidant Anti-inflammatory Anti-apoptotic AchE enhancement (betulin in combination with cyclodextrin enhances spatial memory and lowers β-amyloid, TNF-α, and APLP2 levels)
Zhao et al., 2013 [95]	AlCl_3_ 200 mg/kg 183 days Oral	Ginsenoside Rb1 20 mg/kg 121 days Oral	-	Female ICR mice (60 days old) Weight N/M	Sodium pentobarbital (50 mg/kg)	MWM	Prevent tau hyperphosphorylation Improve learning and memory

Note: N/M: not mentioned. AlCl_3_: aluminum chloride. mg/kg: milligrams per kilogram of body weight. g/kg: grams per kilogram of body weight. μg/kg: micrograms per kilogram of body weight. g: grams. p.p.m.: parts per million. RIV: Rivastigmine. PAT: passive avoidance. MWM: Morris water maze. AAT: active avoidance test. EPM: elevated plus maze. NOR: novel object recognition. RAM: radial arm maze. OFT: open field test. ORM: object recognition memory. FST: forced swim test. LA: locomotor Activity. LEA: locomotor and exploratory activity. RRT: Rotarod test. SDIA: Step-Down Inhibitory Avoidance. HWM: Hebb–Williams maze. BWT: beam walking test. PAMAM: polyamidoamine dendrimers. Lf: lactoferrin-conjugates. AChE: acetylcholinesterase. i.p.: intraperitoneal. i.g.: intragastric. TNF-α: tumor necrosis factor alpha. Aβ: Beta-amyloid. ICR mice: Institute of Cancer Research mice. NMRI mice: Naval Medical Research Institute mice. TCA: tricarboxylic acid. APLP2: amyloid beta precursor-like protein 2. PG: prostaglandin. ES: E synthases. Eps: prostaglandin E receptors. NF-κB: nuclear factor-kappa.

### 3.3. Outcomes of Studies

The outcomes of the included studies, i.e., their behavioral, biochemical, molecular, and histological aspects, were reported to observe the effects of different treatment agents. Behavioral outcomes mainly rely on learning, memory, and motor coordination changes because the observational data show the functional impact of treatments on neurological health. Furthermore, biochemical changes reflect behavioral alterations that include cytokines, anti-oxidant enzymes, and neurotransmitter levels in rodents. Molecular outcomes provide information about gene and protein expression pathways through which treatments can lead to neuroprotection or therapeutic interventions. Histopathological outcomes support these findings by indicating structural alterations within the brain tissue itself, including changes in the neuronal packaging and synaptic connections and decreased neuroinflammation or neurodegeneration. Collectively, these outcomes provide a broad approach to understanding the effectiveness and processes of the treatment agents examined and help to determine potential therapeutic methods. The detailed outcome of each factor is given in Table 3.

**Table 3:** Outcomes of the included studies.

Study	Treatment Agent	Behavioral Outcomes	Biochemical Outcomes	Molecular Outcomes	Histological Results
Abdel-Aal et al., 2011 [38]	Memantine	Improved learning and memory (MWM, RAM, PAT)	N/A	N/A	N/A
Abdel-Aal et al., 2011 [61]	Rivastigmine	Improvement in locomotion and exploration (open field test, Rotarod), enhancement in learning and memory (MWM, RAM, PAT)	N/A	N/A	N/A
Abdel-Aal et al., 2022 [37]	Naproxen	Improved memory and cognitive performance (MWM, NOR, PAT)	↓ hippocampal AChE activity	N/A	Partial restoration of Purkinje cell morphology cerebellar cortex (H and E staining) (IHC)
Abdel-Zaher et al., 2017 [62]	Citicoline	↓ reference and working memory errors (RAM), improved escape latency and swimming (MWM), ↑ step-through latency (PAT)	↓ MDA, ↓ glutamate and ↓ nitrite levels, ↑ GSH levels in the hippocampus	N/A	N/A
Allagui et al., 2014 [63]	Melatonin	Improved cognitive and memory functions (RAM)	↓ TBAR levels ↑ SOD, CAT, GPx, and AchE	N/A	Enhanced cellular structure ↓ vacuolization ↓ cellular depletion ↓ Pyknotic nuclei (H and E staining of hippocampus and neocortex)
Alzahrani et al., 2020 [64]	Azilsartan and Perindopril	Improved spatial working memory (↑ spontaneous alternation %age, Y maze)	↓ AChE, ↓ MDA, and ↓ TNF-α, no significant change in NO, ↓ Aβ_42_ plaques, ↓ neurofibrillary tangles	N/A	Preserved the structure in DG and CA3 hippocampal regions, ↓ vacuolation and ↓ neuronal degeneration (H and E staining)
Amjad and Umesalma, 2015 [65]	*Centella asiatica*	Improved spatial memory, ↓ task latency (Hebb–Williams maze), improved neuromuscular coordination (Rotarod test), improved locomotor activity	↑ MDA levels, ↑ SOD, ↑ CAT, ↑ GST, ↑ GSH, ↓ LPO, SOD, CAT, attenuated AChE levels	N/A	Preserved cell membrane integrity and improved histoarchitecture (in cortex, striatum, hypothalamus, and hippocampus, H and E staining), ↓ Nissl bodies, marked reduction in neuronal cell loss (Nissl’s staining)
Azib et al., 2019 [66]	*Pistacia lentiscus L.* leaves extract	Improved memory (MWM), ↓ anxiety (head dipping, Black and White, EPM)	↓ lipid-peroxidation-maintained SOD and CAT activity, stabilized total thiol levels	N/A	Preserved cerebral cortex morphology, ↓ neuronal loss, and ↓ vacuolation (H and E staining)
Azib et al., 2020 [67]	*Fraxinus angustifolia Vahl.* bark extract	Improved locomotor activity (open field test), ↓ anxiety (Black and White test), improved memory (MWM)	↓ lipid peroxidation, ↑ cell viability in PC12 cells, ↓ MDA in synaptosomes	N/A	Preserved brain cortex structure, reduced vacuolation and neuronal loss (H and E staining)
Bhargava et al., 2023 [14]	*Cassia tora* extract	Improve learning and memory (MWM)	↓ AChE and MAO activities normalized pro-inflammatory markers (↓ IL-6, ↓ IL-1β, ↓ TNF-α, ↓ IFN-γ), amyloid markers (↓ Aβ-42, ↑ Aβ-40) ↓ lipid peroxidation ↑ (CAT, SOD, GSH, GPx, GR, GST)	N/A	Protective effect (in cortex and hippocampus, H and E staining)
Campos et al., 2022 [68]	Chrysin	Improve non-spatial long-term memory (Step-Down Avoidance Test), improved motor function (Chimney Test)	↓ MDA, ↑ SOD, CAT, and GSH levels, ↓ LPO, ↓ AChE activity, ↓ carbonylated protein levels	↓ pro-inflammatory cytokines; ↓ iNOS, ↓ TNFα, ↓ IL-1β in microglial THP-1 cells	Preserved hippocampal and cortical morphology, ↓ vacuolation and pyknosis (H and E staining)
Cao et al., 2017 [69]	*Hypericum perforatum* extract	Improved cognitive function (MWM)	↓ AchE, ↓ glutamic acid, ↑ noradrenaline, and ↑ dopamine ↓ SOD, ↓ GSH, ↑ ROS, ↑ TBARS	↓ IL-6, ↓ IL-1β, ↓ TNF-α, and ↓ MHC class II	↓ Aβ42 and amyloid plaques in the hippocampus (Congo red staining)
Cheng et al., 2014 [70]	Apple (Ralls) polyphenol extract	Improved spatial memory (MWM), preserved cognitive performance (Step-Down Inhibitory Avoidance)	↑ AChE and CK activity, ↑ ATP synthesis, ↓ MDA, ↑ SOD and CAT levels	↓ Aβ accumulation, ↓ neurofibrillary degeneration	Preserved cerebral cortex morphology, ↓ neuronal vacuolation and congestion (H and E staining)
Dibacto et al., 2022 [71]	*Xylopia parviflora*	Improved memory and locomotion (MWM, open field)	↓ AChE and BChE, ↑ SOD, ↑ CAT, ↑ GSH, ↓ MDA, and ↓ NO levels, ATPases activities Assay: ↑ Na^+^, K^+^-ATPase, ↑ Mg^+2^-ATPase activities, ↑ Mg^+2^ levels, and ↓ Ca^+2^ levels	N/A	Improvement of the hippocampal structure (H and E staining)
Firdaus et al., 2022 [21]	*Centella asiatica*	Improved stress and coping behaviors (Y maze) Increased ambulation	↓ MDA and AChE, ↓ SOD and ↓ CAT in cerebrum and cerebellum	N/A	Increase cell count, normalize cell structure H and E staining (cerebrum)
Gadouche et al., 2018 [72]	Pomegranate juice	Improved memory and cognitive function (MWM), ↓ anxiety (EPM), ↓ immobility time (forced swim test)	↓ aluminum accumulation in brain tissue	N/A	Preserved cerebral cortex and hippocampal structure, ↓ neuronal loss and ↓ vacuolization (H and E staining)
García et al., 2009 [60]	Melatonin	No significant results	No significant decrease in aluminum levels in the brain’s hippocampus, cerebellum, and cortex	N/A	N/A
Gong et al., 2005 [73]	*Ginkgo biloba* leaf extract	Improved spatial learning and memory (↓ escape latency and searching distance, MWM)	N/A	N/A	↓ expression of APP and caspase-3 (IHC) Free-floating incubation SABC method
Gothwal et al., 2019 [74]	Rivastigmine	Improved memory and discrimination (ORM)	No significant differences in dopamine or DOPAC	N/A	↓ AChE activity levels, better neuronal integrity (AChE Histo-Enzymology)
Guo et al., 2016 [75]	Misoprostol	Improved spatial learning and memory (MWM)	↓ MDA levels, ↑ SOD	↓ PGE2, mPGES-1, EP2, and EP4, ↑ EP3	↓ hippocampal neuron death restored neuronal structure (H and E staining)
Justin Thenmozhi et al., 2017 [76]	Hesperidin	Improved memory (RAM, EPM, PAT)	↓TBARS ↑ SOD, GSH, CAT, and GPx in brain cortex, hippocampus, and cerebellum	↓ pro-apoptotic (Bax) ↑ anti-apoptotic (Bcl-2)	N/A
Justin-Thenmozhi et al., 2018 [77]	Hesperidin	Improved memory and motor coordination (Y maze, NOR)	↓ Cytosole cytochrome c, caspase-3,8 and 9 levels ↓ GFAP, Iba-1, IL-1β, TNF-α, IL-4 and 6, COX-2, and iNOS	↓ pTau and CDK5 levels (enhanced Aβ clearance through upregulation of IDE, ↑ phosphorylated Akt (pAkt) and GSK-3β)	N/A
Kakkar and Kaur, 2011 [78]	Curcumin (solid lipid nanoparticles of curcumin and free curcumin)	Improved memory (MWM) Improvement was more effective with solid lipid nanoparticles of curcumin than free curcumin	↓ AchE, LPO, SOD, CAT, GSH (curcumin solid lipid nanoparticles more effective)	N/A	Normal neurons with intact nucleus and astrocytes No vacuolization or spongiosis and degenerated neurons (H and E staining of lateral sections of the brain)
Kumar et al., 2019 [79]	Artesunate + Rivastigmine	Improved learning and memory (PAT)	N/A	N/A	Protective effect on brain histology (H and E staining)
Li et al., 2018 [80]	Isorhynchophylline	Improved learning and memory (RAM)	↑ SOD, ↓ MDA, ↑ CAT ↑ GSH, ↓ ACHE, and no significant effect on BuChE	↓ phosphorylation of IκBα and NF-κB p65	N/A
Liu et al., 2010 [81]	Soy isoflavones	Improved learning and memory (PAT)	↓ ACHE ↑ glutamic acid and aspartic acid levels in cortex and hippocampus	N/A	N/A
Luo et al., 2007 [82]	Icariin	Improved spatial memory (↓ escape latency and searching distance, ↑ exploring time in the target area, MWM)	↑ SOD, ↓ MDA in the hippocampus	N/A	Attenuated Aβ_1-40_ levels in the hippocampus (IHC), free-floating incubation and SABC method
Mohamed et al., 2023 [83]	*Echinacea purpurea* extract	Improved spatial and short-term memory (Y maze), improved long-term memory (NOR) and depressive-like behaviors (forced swim)	Inhibited Acholinesterase restored oxidative balance	↓ IL-6 and TNF-α cytokines	Normalize cell structure, no amyloid plaques in cortex (H and E and Congo red staining), improved histology (CA 3,4 and DG region of hippocampus, H and E staining)
Nampoothiri et al., 2017 [84]	Insulin	Memory improvement not observed (MWM)	↓ blood glucose ↑ AChE, and the results were not significant compared to AlCl_3_ ↑ GSH in brain hippocampus and frontal cortex, showing oxidative stress	N/A	N/A
Nehru and Bhalla, 2007 [85]	Centrophenoxine	Improved memory (active and passive avoidance)	Restored AChE activity (up to 93% increase in the cerebrum) Restored levels of DA, norepinephrine, and serotonin in all brain regions	N/A	N/A
Pan et al., 2015 [86]	Beraprost sodium	Improved cognitive and memory functions (MWM)	↓ MDA and restored SOD activity ↓ 6-k-PGF_1α_ levels	↓ PGIS mRNA expression ↓ IP mRNA expression	Improved histopathological changes of hippocampal neurons (H and E staining)
Prabhakar, 2020 [87]	Naringin	Improved locomotor and exploratory activity, ↓ latency time (MWM), ↑ memory performance (MWM RAM)	↓ AChE, ↓ MDA, ↑ SOD, and ↑ CAT levels	N/A	N/A
Qi-Hai et al., 2006 [88]	*Ginkgo biloba* leaf extract	Improved spatial memory, ↓ escape latency and search distance (MWM)	N/A	N/A	Reversed AChE levels to near-normal in high doses, ↓ AChE expression (in high dose of extract) (free-floating staining and SABC method)
Rapaka et al., 2021 [89]	*Benincasa hispida*	Improved memory (MWM, Y maze)	↑ AchE, DA, and serotonin levels in the cortex and hippocampus ↓ MDA, ↑ SOD and GSH, ↓ TNF-α, and ↓ IL-1β	↑ Nrf2/HO-1 pathway, showing enhanced anti-oxidant defense	Histopathological examination revealed preserved neuronal structure, ↓ amyloid plaques and neurodegeneration (H and E staining of hippocampal CA3)
Ravi et al., 2018 [20]	*Caesalpinia crista* methanolic extract	Improved spatial memory (MWM)	↓ AChE activity restored anti-oxidant enzyme levels (CAT, GSH, GST), ↓ MDA	↓ pro-inflammatory cytokines (IL-6, TNF-α, IL-1β); ↑ BDNF levels	Improved cell morphology, partial restoration of morphology of Purkinje cells ↑ GFAP, ↓ caspase-3, and ↑ Nestin expression (IHC) (H and E staining) Hippocampus and cerebellum
Ravi et al., 2020 [90]	*Cassia tora* extract	Improve spatial memory (MWM) Restore motor activity	↓ Tthioflavin-T fluorescence, ↓ Aβ_1–42_ aggregation ↑ CAT, GPx, GST ↓ MDA, ↓ AChE ↓ TNF-α, IL-6, IL-1β mRNA levels ↑ BDNF level	N/A	↑ Density of mature Pyramidal cell ↓ Pyknotic neurons: cytoarchitecture: ameliorate pyramidal cell layer alignment (Cresyl violet hippocampal CA3 and CA1)
Rijal et al., 2019 [91]	Phenylpropanoids; para-methoxycinnamic acid and ethyl-p-methoxycinnamate	Improved spatial memory, ↓ escape latency (MWM)	Restored CAT and ↓ TBARS levels in the hippocampus and cortex, ↓ hippocampal AChE activity	N/A	N/A
Saba et al., 2017 [23]	Rasa Sindoor	Improved memory (MWM)	↑ glutamate, GABA, and aspartate in cortex, hippocampus, and striatum	N/A	Aβ plaques in aluminum-treated group (IHC of cortex and hippocampus of aluminum and control group only)
Sethi et al., 2009 [36]	Curcumin	Anxiety ↓ (open field) Memory performance improved (MWM)	Cytosolic PKC activity: ↓ in young; ↑ in old Bound PKC activity: maintained, lipid peroxidation ↓ Na–K-ATPase activity ↑	N/A	Normal cytoplasm and intact cellular organelles (histological assessment using transmission electron microscopy of cortical cells)
Shalaby et al., 2023 [92]	Ginsenoside Rb1	Enhanced memory, ↑ latency into the dark chamber (PAT)	↓ MDA, ↑ SOD, ↓ AChE levels, ↓ Aβ _40_ and phosphorylated tau	N/A	Restoration of normal neuronal architecture, ↓ shrinkage and vacuolation (H and E staining), ↑ surviving neurons (Nissl’s staining), ↑ synaptophysin, ↓ cleaved caspase-3, Iba-1 ↓ GFAP ↓ expression (IHC)
Singh et al., 2018 [24]	EGCG (Epigallocatechin-gallate) and EGCG-loaded nanoparticles	↑ locomotor activity (open field) ↑ recognition memory (NOR) ↑ spatial and working memory (MWM)	↓ AChE ↓ ROS ↓ NO	↓ Aβ_1–42_, AChE, APP, and GSK3β ↑ PDK1	↓ reduced neurofibrillary tangles ↓ Aβ_1–42_ (H and E staining of cortex and hippocampus)
Thenmozhi et al., 2016 [93]	Tannoids of *Emblica officinalis*	Improved reference and working memory (RAM), improved cognitive performance (EPM, PAT)	↓ lipid peroxidation (TBARS) and ↑ GSH, ↑ SOD, ↑ CAT, ↑ GPx levels in the hippocampus, cortex, and cerebellum	↓ Bax, ↓ caspase -3 and 9, ↓ cytosolic cytochrome c, and ↑ Bcl-2 and ↑ mitochondrial cytochrome c expression	N/A
Thirunavukkarasu et al., 2012 [94]	Manasamitra vatakam	Improved memory performance (active-avoidance), ↓ anxiety (EPM)	↑ AChE, SOD, CAT, GPx, and GSH levels; ↓ LPO	↓ aluminum concentration in the cortex and hippocampus, ↓ HSP70 protein and mRNA expression	↓ neuronal shrinkage, vacuolated cytoplasm cellular depletion and necrosis (cerebral cortex and hippocampus, H and E staining)
Zakrzeska et al., 2023 [59]	Betulin	Improved spatial memory (MWM), improved motor coordination (beam walking test)	↓ Aβ_1-42_, ↓ TNF-α and ↓ APLP2 levels, ↑ AChE activity, ↑ anti-oxidant enzymes (GSH, GR, GSTs), ↓ oxidative stress markers, ↓ GSSG, ↓ G6PD, and ↓ GPO	N/A	N/A
Zhao et al., 2013 [95]	Ginsenoside Rb1	Improved learning and memory (MWM)	N/A	Reduced tau phosphorylation (by restoring levels of ↓ active p-GSK3 and ↑ PP2A) in the cortex and hippocampus	↓ p-tau staining, restore p-GSK3 and PP2A (in hippocampal CA3, IHC)

Note: N/A: not applicable. ↓ decrease. ↑ increase. PAT: passive avoidance test. EPM: elevated plus maze. MWM: Morris water maze. RAM: radial arm maze. NOR: novel object recognition. ORM: object recognition memory. AChE: acetylcholinesterase. BChE: Butyrylcholinesterase. MDA: Malondialdehyde. SOD: superoxide dismutase. CAT: catalase. GST: Glutathione S-Transferase. GSH: Reduced Glutathione. GPx, GPO: Glutathione Peroxidase. GR: Glutathione Reductase. GSSG: Glutathione disulphide. ROS: reactive oxygen species. LPO: lipid peroxidation. IL-1β: Interleukin-1 beta. IL-4: Interleukin-4. IL-6: Interleukin-6. COX-2: cyclooxygenase-2. iNOS: Inducible Nitric Oxide Synthase. PKC: Protein Kinase C. NO: Nitric Oxide. BDNF: Brain-Derived Neurotrophic Factor. CDK5: Cyclin-Dependent Kinase 5. TNF-α: tumor necrosis factor alpha. Aβ: Beta-amyloid. TCA: tricarboxylic acid. APLP2: amyloid beta precursor-like protein 2. PG: prostaglandin. ES: E synthases. Eps: prostaglandin E receptors. NF-κB: nuclear factor-kappa B. IκBα: inhibitor of nuclear factor-kappa B. Iba-1: ionized calcium-binding adaptor molecule 1. GFAP: glial fibrillary acidic protein. IDE: Insulin-Degrading Enzyme. pAkt: Phosphorylated Protein Kinase B. GSK: Glycogen Synthase Kinase. 6-k-PGF1α: 6-keto prostaglandin F1α. TBARS: Thiobarbituric Acid Reactive Substances. G6PD: Glucose-6-Phosphate Dehydrogenase. Bcl-2: B-cell lymphoma 2. PDK1: Phosphoinositide-Dependent Kinase 1. GABA: Gamma-Aminobutyric Acid. APP: Amyloid Precursor Protein. IFN-γ: Interferon-Gamma. MHC II: major histocompatibility complex class II. PDK1: 3-Phosphoinositide-dependent kinase 1. DOPAC: 3, 4-Dihydroxyphenylacetic acid. MAO: platelet monoamine oxidase. SABC: Streptavidin-Biotin Amplification Complex. CA: Cornu Ammonis. DG: Dentate Gyrus. H and E staining: Hematoxylin and Eosin staining. IHC: immunohistochemistry.

### 3.4. Behaviors to Access Learning and Memory

In rodent models, to assess learning and memory, different behaviors are performed with tasks that are designed specially to evaluate different cognitive domains. In 45 extracted studies, 16 different behaviors were assessed. The details of behaviors reported in the shortlisted studies are mentioned in Table 4. MWM is used to evaluate spatial learning and memory in laboratory animals, specifically rodents. The ability of rodents to find an obscure platform in a water-filled arena is measured [96]. It measures hippocampal-dependent learning and memory through parameters like latency and distance travelled to the platform [97]. The Y maze measures spatial working memory in rodents, in which they are allowed to choose between two arms without exposing them to stressors to assess their ability to remember previous choices [98]. The radial arm maze is used to evaluate spatial learning and memory in rodents, which measures performance through errors and navigation strategies across multiple trials [99]. The open field test evaluates exploration, locomotion, and anxiety-like behaviors in rodents [100]. It is adapted for specific cognitive tasks like object recognition, where the memory of rodents is assessed based on their exploration of novel objects [101]. The passive avoidance task is used to evaluate the learning and memory of rodents by measuring their ability to avoid an aversive stimulus, which will indicate their memory retention and cognitive function [102]. In the active avoidance task, the decision-making, learning, and memory capabilities of rodents are assessed based on their ability to avoid shock or aversive stimulus through behavioral response towards the stimulus [103]. In object recognition memory, rodents’ ability to recognize familiar or novel objects over time is measured [104].

**Table 4:** List of behaviors performed in the selected studies.

Behavior Name	Number of Studies in Which Behavior Is Assessed
Morris water maze	29
Open field	12
Passive avoidance task	10
Elevated plus maze	10
Radial arm maze	8
Locomotor activity	5
Novel object recognition	4
Y maze	4
Object recognition memory	2
Rota rod	2
Active avoidance test	2
Forced swim	2
Step-down inhibitory avoidance	2
Black and White test	2
Hebb–Williams maze	2
Head dipping	1

### 3.5. The Methodological Quality of the Included Studies

All of the studies were published in peer-reviewed journals, and the majority of the studies reported animal sex (97.7%), animal strain (100%), and sample size (100%). Furthermore, 82% of the studies mentioned conditions; housing temperature was reported by 34 studies, and standard conditions were reported by 3 studies. Random allocation to groups was mentioned in 20 (44%) studies. Meanwhile, 27 studies (60%) did not mention which anaesthetic agent they used, while 9 studies (20%) used an anaesthetic agent with neuroprotective properties (e.g., ketamine, sodium pentobarbital, barbitone, thiopental sodium, etc.) and 8 studies (18%) used an anaesthetic agent that does not possess any neuroprotective properties. The potential conflict of interest statement was mentioned in 37 studies (82%). The methodological quality of the studies is shown in Table 5.

**Table 5:** Methodological quality of the included studies.

Study	A	B	C	D	E	F	G	H	Total 8
Abdel-Aal et al., 2011 [38]	✓	☓	✓	✓	✓	?	✓	☓	5
Abdel-Aal et al., 2011 [61]	✓	☓	✓	✓	✓	?	✓	☓	5
Abdel-Aal et al., 2022 [37]	✓	✓	☓	✓	✓	☓	✓	✓	6
Abdel-Zaher et al., 2017 [62]	✓	☓	✓	✓	✓	?	✓	✓	6
Allagui et al., 2014 [63]	✓	☓	✓	✓	✓	?	✓	✓	6
Alzahrani et al., 2020 [64]	✓	✓	✓	✓	✓	?	✓	✓	7
Amjad and Umesalma, 2015 [65]	✓	☓	✓	✓	✓	✓	✓	☓	6
Azib et al., 2019 [66]	✓	☓	✓	✓	✓	?	✓	✓	6
Azib et al., 2020 [67]	✓	☓	✓	✓	✓	?	✓	✓	6
Bhargava et al., 2023 [14]	✓	✓	✓	✓	✓	☓	✓	✓	7
Campos et al., 2022 [68]	✓	✓	✓	✓	✓	☓	✓	✓	7
Cao et al., 2017 [69]	✓	✓	✓	✓	✓	☓	✓	✓	7
Cheng et al., 2014 [70]	✓	✓	✓	✓	✓	?	✓	✓	7
Dibacto et al., 2022 [71]	✓	☓	✓	✓	✓	?	✓	✓	6
Firdaus et al., 2022 [21]	✓	☓	✓	✓	✓	?	✓	✓	6
Gadouche et al., 2018 [72]	✓	☓	✓	✓	✓	?	✓	✓	6
García et al., 2009 [60]	✓	☓	✓	✓	✓	☓	✓	✓	6
Gong et al., 2005 [73]	✓	☓	✓	✓	✓	✓	✓	✓	7
Gothwal et al., 2019 [74]	✓	☓	☓	✓	✓	?	✓	✓	5
Guo et al., 2016 [75]	✓	✓	☓	✓	✓	?	✓	✓	6
Justin Thenmozhi et al., 2017 [76]	✓	✓	✓	✓	✓	?	✓	✓	7
Justin-Thenmozhi et al., 2018 [77]	✓	✓	☓	✓	✓	☓	✓	✓	6
Kakkar and Kaur, 2011 [78]	✓	☓	✓	✓	✓	?	✓	✓	6
Kumar et al., 2019 [79]	✓	✓	✓	✓	✓	?	✓	✓	7
Li et al., 2018 [80]	✓	✓	✓	✓	✓	?	✓	✓	7
Liu et al., 2010 [81]	✓	✓	✓	✓	✓	?	✓	✓	7
Luo et al., 2007 [82]	✓	☓	✓	✓	✓	?	✓	☓	5
Mohamed et al., 2023 [83]	✓	☓	✓	✓	✓	☓	✓	✓	6
Nampoothiri et al., 2017 [84]	✓	☓	✓	✓	✓	?	✓	✓	6
Nehru and Bhalla, 2007 [85]	✓	✓	☓	✓	✓	?	✓	☓	5
Pan et al., 2015 [86]	✓	✓	✓	✓	✓	✓	✓	✓	8
Prabhakar, 2020 [87]	✓	☓	✓	✓	✓	?	✓	✓	6
Qi-Hai et al., 2006 [88]	✓	☓	✓	✓	✓	✓	✓	☓	6
Rapaka et al., 2021 [89]	✓	☓	✓	✓	✓	✓	✓	✓	7
Ravi et al., 2018 [20]	✓	✓	✓	✓	✓	?	✓	✓	7
Ravi et al., 2020 [90]	✓	✓	✓	✓	✓	☓	✓	✓	7
Rijal et al., 2019 [91]	✓	☓	☓	✓	✓	?	✓	✓	5
Saba et al., 2017 [23]	✓	☓	✓	✓	✓	✓	✓	✓	7
Sethi et al., 2009 [36]	✓	✓	✓	✓	✓	☓	✓	☓	6
Shalaby et al., 2023 [92]	✓	✓	✓	✓	✓	✓	✓	✓	8
Singh et al., 2018 [24]	✓	✓	✓	✓	✓	?	✓	✓	7
Thenmozhi et al., 2016 [93]	✓	☓	☓	✓	✓	?	✓	☓	4
Thirunavukkarasu et al., 2012 [94]	✓	☓	✓	✓	✓	?	✓	✓	6
Zakrzeska et al., 2023 [59]	✓	☓	✓	☓	✓	✓	✓	✓	6
Zhao et al., 2013 [95]	✓	✓	☓	✓	✓	☓	✓	✓	6

Note: Studies that meet the following criteria. A: Peer-reviewed publication? B: Were animals randomly allocated in groups? C: Is the housing temperature reported? D: Is the sex of the animals reported? E: Is the strain of the animals reported? F: Avoidance of anaesthetic agents with neuroprotective properties. G: Sample size mentioned. H: Potential conflict of interests statement. ✓ = fulfil the criterion, ☓ = do not fulfil the criterion, = not mentioned, ? = anaesthetic agent not mentioned in the study.

### 3.6. Transparency and Reporting Standards of Studies

Methodological quality ratings of the selected studies varied from 9 to 15 out of a possible 15 points, with 12 serving as the median. The dosage and duration of aluminum and treatment agents are mentioned in all of the studies. Models are validated in 100% of studies. Animal ethical approval or compliance with animal welfare guidelines was reported by 44 studies (97.7%), toxicity of treatment agents was mentioned by 12 studies (26.6%), 18 studies mentioned the euthanasia protocol used for anaesthetizing animals (40%), and 20 studies reported all four characteristics (sex, strain, weight, age) of animals (44.4%). All three histology, biochemical, molecular assessment factors were reported by 10 studies (22.2%), and more than three behaviors were reported by 16 studies (35.5%). The Transparency and Reporting Standards of the studies are assessed in Table 6.

**Table 6:** Transparency and Reporting Standards of studies.

Study	(1)	(2)	(3)	(4)	(5)	(6)	(7)	(8)	(9)	(10)	Total Score 15
Abdel-Aal et al., 2011 [38]	1	1	1	1	0	1	0	4	0	1	10
Abdel-Aal et al., 2011 [61]	1	1	1	1	0	1	0	4	0	1	10
Abdel-Aal et al., 2022 [37]	1	1	1	1	0	1	1	3	2	1	12
Abdel-Zaher et al., 2017 [62]	1	1	1	1	0	1	0	3	1	1	10
Allagui et al., 2014 [63]	1	1	1	1	0	1	0	3	2	1	11
Alzahrani et al., 2020 [64]	1	1	1	1	0	1	0	4	2	0	11
Amjad and Umesalma, 2015 [65]	1	1	1	1	1	1	1	3	2	1	13
Azib et al., 2019 [66]	1	1	1	1	1	1	0	3	2	1	12
Azib et al., 2020 [67]	1	1	1	1	1	1	0	3	2	1	12
Bhargava et al., 2023 [14]	1	1	1	1	0	1	1	3	2	0	11
Campos et al., 2022 [68]	1	1	1	1	1	1	1	4	3	1	15
Cao et al., 2017 [69]	1	1	1	1	0	1	1	4	3	0	13
Cheng et al., 2014 [70]	1	1	1	1	0	1	0	4	3	0	12
Dibacto et al., 2022 [71]	1	1	1	1	1	1	0	4	2	0	12
Firdaus et al., 2022 [21]	1	1	1	1	0	1	0	3	2	0	10
Gadouche et al., 2018 [72]	1	1	1	1	0	1	0	4	2	1	12
García et al., 2009 [60]	1	1	1	1	0	1	1	3	1	0	10
Gong et al., 2005 [73]	1	1	1	1	0	1	1	4	1	0	11
Gothwal et al., 2019 [74]	1	1	1	1	0	1	0	3	2	0	10
Guo et al., 2016 [75]	1	1	1	1	0	1	0	3	3	0	11
Justin Thenmozhi et al., 2017 [76]	1	1	1	1	0	1	0	4	2	1	12
Justin-Thenmozhi et al., 2018 [77]	1	1	1	1	1	1	1	4	2	1	14
Kakkar and Kaur, 2011 [78]	1	1	1	1	0	1	0	4	2	0	11
Kumar et al., 2019 [79]	1	1	1	1	0	1	0	3	1	0	9
Li et al., 2018 [80]	1	1	1	1	1	1	0	4	2	0	12
Liu et al., 2010 [81]	1	1	1	1	0	1	0	3	1	0	9
Luo et al., 2007 [82]	1	1	1	1	1	1	0	3	2	0	11
Mohamed et al., 2023 [83]	1	1	1	1	1	1	1	3	3	1	14
Nampoothiri et al., 2017 [84]	1	1	1	1	0	1	0	4	1	0	10
Nehru and Bhalla, 2007 [85]	1	1	1	1	0	1	0	3	1	0	9
Pan et al., 2015 [86]	1	1	1	1	0	1	1	4	3	0	13
Prabhakar, 2020 [87]	1	1	1	1	0	1	0	3	1	1	10
Qi-Hai et al., 2006 [88]	1	1	1	1	0	0	1	4	1	0	10
Rapaka et al., 2021 [89]	1	1	1	1	1	1	1	4	3	0	14
Ravi et al., 2018 [20]	1	1	1	1	0	1	0	3	3	0	11
Ravi et al., 2020 [90]	1	1	1	1	0	1	1	3	2	0	11
Rijal et al., 2019 [91]	1	1	1	1	0	1	0	4	1	0	10
Saba et al., 2017 [23]	1	1	1	1	0	1	1	3	2	0	11
Sethi et al., 2009 [36]	1	1	1	1	0	1	1	3	2	0	11
Shalaby et al., 2023 [92]	1	1	1	1	0	1	1	4	2	0	12
Singh et al., 2018 [24]	1	1	1	1	0	1	0	3	3	1	12
Thenmozhi et al., 2016 [93]	1	1	1	1	0	1	0	4	2	1	12
Thirunavukkarasu et al., 2012 [94]	1	1	1	1	1	1	0	3	3	0	12
Zakrzeska et al., 2023 [59]	1	1	1	1	1	1	1	2	1	0	10
Zhao et al., 2013 [95]	1	1	1	1	0	1	1	3	2	0	11

(1) Aluminum dose mentioned, (2) treatment dose mentioned, (3) duration of treatment mentioned, (4) model validation, (5) toxicity of agent evaluated, (6) compliance with animal welfare regulations or animal ethical approval, (7) euthanasia protocol mentioned, (8) demographic data (sex, strain, weight, age) (1 for each trait), total of 4, (9) factors assessed (histology, biochemical, molecular assessment), 1 for each, (10) number of behaviors assessed, ≥3 = 1, ≤2 = 0, 1 = mentioned, 0 = not mentioned.

### 3.7. Morris Water Maze Analysis

Research on humans [105] and animals has linked aluminum exposure to learning and memory problems. Epidemiological studies have linked drinking water containing aluminum to AD [8,106,107]. Aluminum neurotoxicity could have a significant impact on the emergence of depression [108], anxiety disorders [109], and memory impairment [110]. Rats treated with aluminum exhibited poor spatial working memory in MWM due to the deterioration of the function of the hippocampus after aluminum exposure [25]. MWM is widely used to evaluate learning and memory [111]. MWM is a behavioral test in which a large circular pool or tank is filled with water, and animals, often rats or mice, are required to escape onto a hidden platform, the position of which can typically only be determined through spatial memory [112]. Escape latency refers to the time taken by the subject to reach the non-visible platform placed below the water in a circular water-filled tank in the probe trial [113]. Escape latency is a key behavioral parameter used to assess learning and memory. Shorter escape latency indicates better spatial learning and memory [111]. A random effect model was selected [53,54].

To perform a meta-analysis, escape latency data from MWM are reported. Among 45 studies, 29 studies performed the MWM test, with 28 measuring escape latency. Other factors were also measured in the studies, like time spent in the target quadrant or exploration time, total distance travelled, and the number of platform crossings. Escape latency was the common factor in all studies, so it was selected for meta-analysis. Among these 29 studies, 21 involved rat models and 7 used mouse models. For analysis using various animal models, the data are categorized into three subgroups, rats, mice, and transgenic mice, to lessen the heterogeneity between groups.

**Figure 2:**
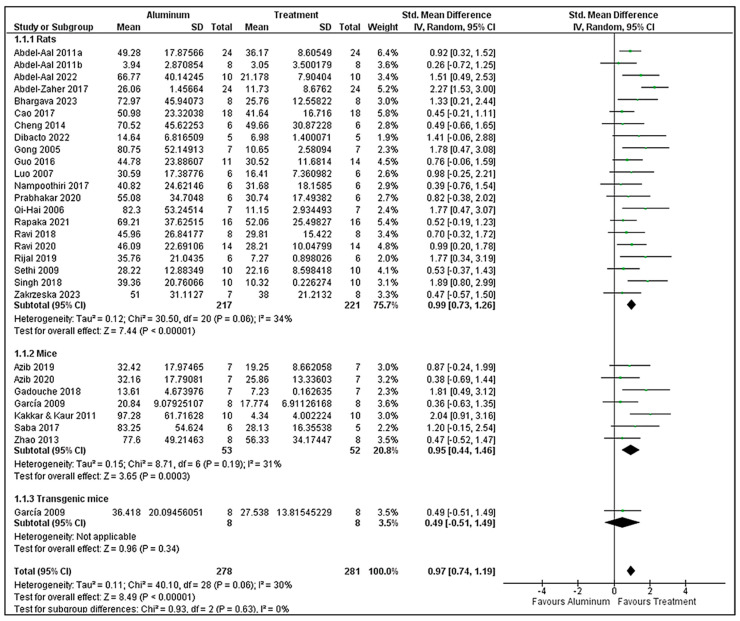
Comparison of 28 studies in a forest plot: aluminum group against treatment group. Outcome: escape latency (sec) of MWM. CI: confidence interval; IV: independent variable; SMD: standardized mean difference.

A total of 273 animals were included in the treatment group (aluminum + treatment agent). Among the studies that used different doses for treatment, the highest dose was selected for the forest plot. In total, 270 animals were added as a positive control, the aluminum group. Escape latency (seconds) was used in the correlation between aluminum and the treatment group because it represents the difference in memory function between them. The forest plot showed that all of the treatments improved escape latency. The meta-analysis revealed a statistically significant pooled effect favoring the treatment over aluminum (SMD = 0.97, 95% CI: 0.74 to 1.19; *p* < 0.00001). Overall heterogeneity was low to moderate, and it was I^2^ = 30%, Tau^2^ = 0.11, and Q stats Chi^2^ = 40.10 (df = 28; *p* = 0.06). This means that while there is some heterogeneity, it is not statistically significant, and the results are consistent between studies. Within the rat subgroup, the pooled effect was significant, as well (SMD = 0.99, 95% CI: 0.73 to 1.26; *p* < 0.00001), and it had low to moderate heterogeneity (I^2^ = 34%), Tau^2^ = 0.12, and Chi^2^ = 30.50 (df = 20; *p* = 0.06). This implies mild inconsistency between studies, possibly owing to differences in study design, sample size, or methods of measurement. For the mouse subgroup, the impact was still statistically significant (SMD = 0.95, 95% CI: 0.44 to 1.46; *p* = 0.0003), with comparable heterogeneity estimates: I^2^ = 31%, Tau^2^ = 0.15, and Chi^2^ = 8.71 (df = 6; *p* = 0.19). Variability is slightly greater here, though still acceptable. The transgenic mouse subgroup was analyzed separately due to their genetically modified status, which may influence biological responses differently than standard animal models. Notably, the transgenic mouse subgroup showed a small, non-significant effect, presumably because it incorporated only a single study, so Tau^2^ did not apply (SMD = 0.49 [−0.51 to 1.49]; *p* = 0.34), and no heterogeneity (Tau^2^ not applicable). The subgroup difference test was not significant (Chi^2^ = 0.93, df = 2; *p* = 0.63; I^2^ = 0%), showing no statistically significant difference in effect size between species (Figure 2). The 95% prediction interval ranges from 0.253 to 1.687 (with standard deviation of 0.349), indicating that while the average effect size is large, future studies may observe moderate to even larger effects, further supporting the consistency and relevance of the treatment effect.

### 3.8. Superoxide Dismutase Analysis

SOD is a major anti-oxidant enzyme that protects cells from oxidative stress caused by superoxide radicals [114]. SOD is distributed in neurons and reactive glial cells, including astrocytes of the brain and spinal cord of both humans and rodents. Brain-specific SODs are crucial for maintaining the redox equilibrium and shielding the brain from oxidative damage. SODs are intracellularly and extracellularly expressed in neurons and glial cells across the central nervous system [115]. The brain’s hippocampus and cerebral cortex, which are areas known to regulate learning and memory, undergo oxidative damage as a result of oxidative stress [116]. Optical microscope analysis of the brain regions of animals in experiments has shown damage and distinctive morphological alterations to the cortex and hippocampal regions, including neurofibrillary neurodegeneration. This damage may be brought on by aluminum buildup in these areas, affecting learning and memory [25]. A random effect model was selected [53,54].

**Figure 3:**
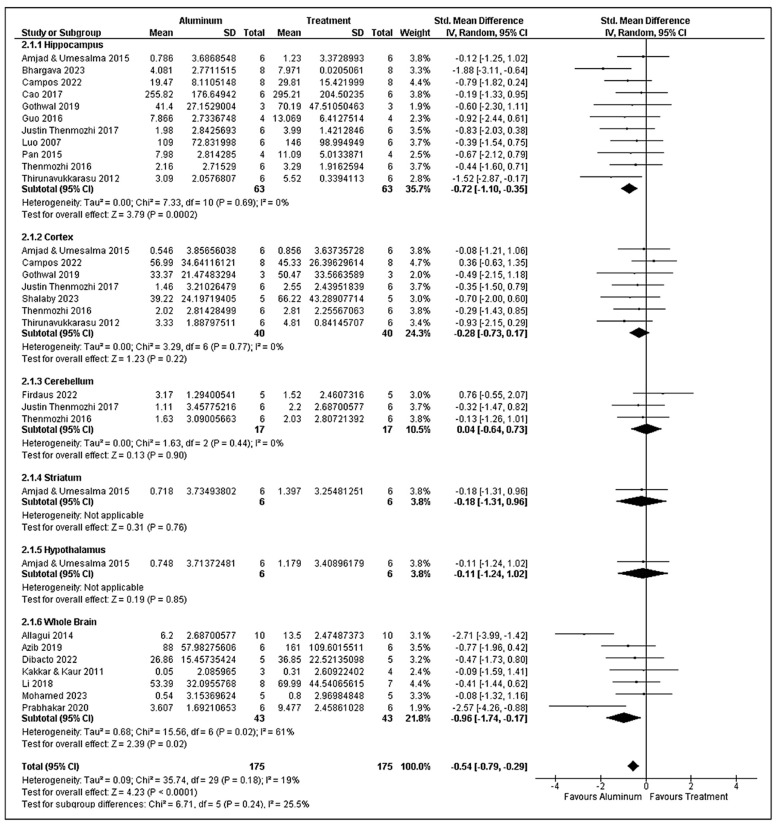
Comparison of 21 studies in a forest plot: aluminum group against treatment group. Outcome: SOD levels. CI: confidence interval; IV: independent variable; SMD: standardized mean difference.

A total of 21 studies estimated SOD levels. A total of 116 animals were included in the treatment group (aluminum + treatment group). Among the studies that used different doses for treatment, the highest dose was selected for the forest plot, except for [66], whose lower dose was chosen as it is more effective. In total, 116 animals were added to the positive control aluminum group. The forest plot showed that all of the treatments increased SOD levels in the treatment group. The subgroup meta-analysis showed region-specific differences in the effects of treatment compared to aluminum exposure. In the hippocampus, a statistically significant pooled effect favored treatment (SMD = −0.72, 95% CI: −1.10 to −0.35; *p* = 0.0002), with no heterogeneity observed (I^2^ = 0%, Tau^2^ = 0.00; Chi^2^ = 7.33, df = 10, *p* = 0.69), indicating consistency across studies. The whole brain subgroup also showed a significant benefit of treatment (SMD = −0.96, 95% CI: −1.74 to −0.17; *p* = 0.02), although moderate heterogeneity was present (I^2^ = 61%, Tau^2^ = 0.68; Chi^2^ = 15.66, df = 6, *p* = 0.02), suggesting some variability between studies. Other brain regions, including the cortex (SMD = −0.28, 95% CI: –0.73 to 0.17; *p* = 0.22), cerebellum (SMD = 0.04, 95% CI: −0.64 to 0.73; *p* = 0.90), striatum (SMD = −0.18, 95% CI: −1.31 to 0.96; *p* = 0.76), and hypothalamus (SMD = −0.11, 95% CI: −1.24 to 1.02; *p* = 0.85) did not show statistically significant effects, as the confidence intervals crossed zero. These subgroup analyses were also marked by low or negligible heterogeneity, as indicated by I^2^ = 0% and Tau^2^ = 0.00 in most cases. This may be because of fewer included studies. The overall pooled effect across all included brain regions favored treatment significantly (SMD = −0.54, 95% CI: −0.79 to −0.29; *p* < 0.0001), with low heterogeneity overall (I^2^ = 19%, Tau^2^ = 0.09; Chi^2^ = 35.74, df = 29; *p* = 0.18). The test for subgroup differences (Chi^2^ = 6.71, df = 5, *p* = 0.24) was not statistically significant, implying that although some brain areas (like the hippocampus and the whole brain) showed stronger effects, the differences between subgroups were not large enough to confirm a statistically significant variation in effect sizes (Figure 3). In future similar studies, the true effect size is expected to fall between −1.203 and 0.123. The standard deviation of the prediction interval is 0.324.

### 3.9. Catalase Analysis

CAT is an important anti-oxidant enzyme that breaks down hydrogen peroxide into water and oxygen and, as a result, protects the cells from oxidative stress [114]. CAT is present in the brain. The strongest CAT activity was found in the substantia nigra and hypothalamus of adult rat brains. Intermediate activity was found in the corpus callosum. The frontal brain and caudate-putamen had the least activity. Although there is significant activity in regions with few microperoxisomes, regional CAT has some correlation with the reported distribution of microperoxisomes. One of the mechanisms that detoxifies H_2_O_2_ produced during CNS amine metabolism may be CAT [117]. The brain’s hippocampus and/or cerebral cortex, which are areas known to regulate learning and memory, undergo oxidative damage as a result of oxidative stress [116]. Optical microscope analysis of the brains of experimental animals has shown damage and distinctive morphological alterations to the cortex and hippocampal regions, including neurofibrillary neurodegeneration. This damage may be brought on by aluminum buildup in these areas and affect learning and memory [25]. A random effect model was selected [53,54].

**Figure 4:**
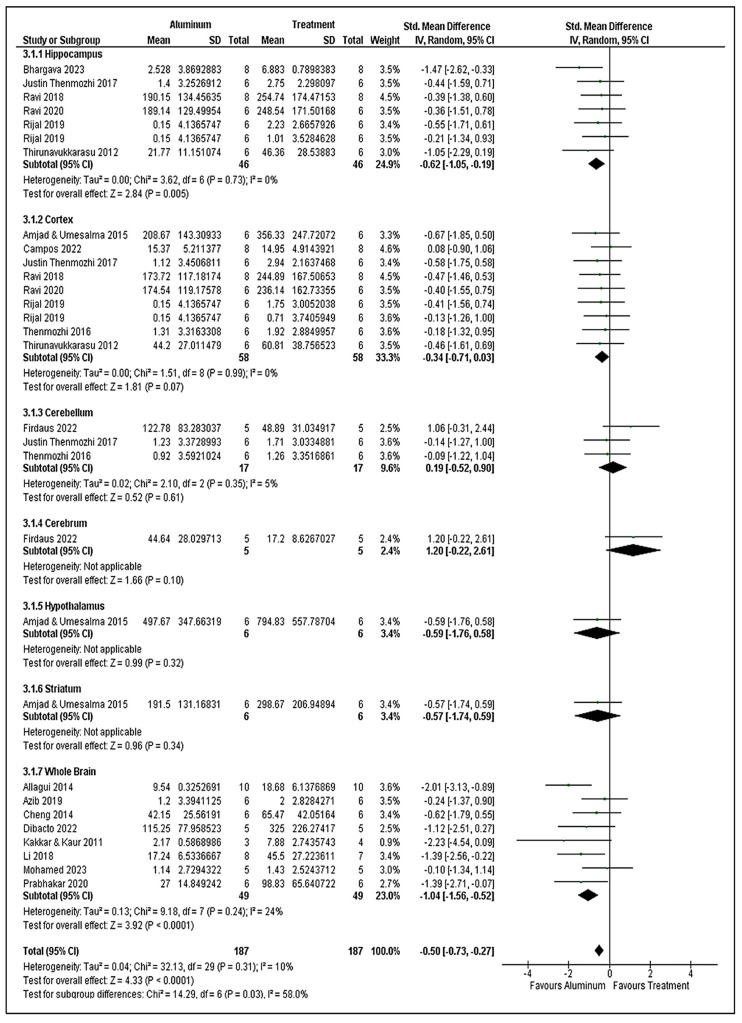
Comparison of 18 studies in a forest plot: aluminum group against treatment group. Outcome: CAT levels. CI: confidence interval; IV: independent variable; SMD: standardized mean difference.

A total of 18 studies estimated CAT levels. A total of 114 animals were included in the treatment group (aluminum + treatment). Among the studies that used different doses for treatment, the highest dose was selected for the forest plot, except for [66], whose lower dose was chosen as it was more effective. In total, 114 animals were added to the positive control aluminum group. The forest plot showed that all treatments increased CAT levels in the treatment group. The meta-analysis revealed a statistically significant small to moderate effect in favor of the treatment relative to aluminum exposure (SMD = −0.50, 95% CI: −0.73 to −0.27; *p* < 0.0001). This indicates that treatment considerably alleviated aluminum-induced neurotoxicity across studies. Heterogeneity between included studies was low (I^2^ = 10%), with Tau^2^ = 0.04 and a Q-statistic (Chi^2^) of 32.13 (df = 29; *p* = 0.31). These findings suggest that the majority of the heterogeneity is probably ascribable to chance rather than actual differences between studies. Subgroup analysis of various brain regions was performed. In the hippocampus subgroup, the combined effect size was statistically significant (SMD = −0.62, 95% CI: −1.05 to −0.19; *p* = 0.005) without heterogeneity (Tau^2^ = 0.00; I^2^ = 0%), and Chi^2^ = 3.62 (df = 6; *p* = 0.73). These findings suggest a homogeneous treatment effect across studies aiming at the hippocampus. In the subgroup of the cortex, the effect of treatment was not statistically significant (SMD = −0.34, 95% CI: −0.71 to 0.03; *p* = 0.07). Heterogeneity is not applicable (Tau^2^ = 0.00; I^2^ = 0%), with a Chi^2^ of 1.51 (df = 8; *p* = 0.99). For the cerebellum, there was a large and significant treatment effect (SMD =0.19, 95% CI: −0.52 to 0.90, *p* = 0.61), with no heterogeneity (Tau^2^ = 0.02; I^2^ = 5%) and Chi^2^ = 2.10 (df = 2; *p* = 0.35). This shows a large and consistent neuroprotective effect in this area. The hypothalamus subgroup was also not significantly affected (SMD = −0.59, 95% CI: −1.76 to 0.58; *p* = 0.32). Heterogeneity statistics are not applicable because there were limited data. The striatum subgroup also did not have a significant effect (SMD = −0.57, 95% CI: −1.74 to 0.59; *p* = 0.34) and did not have heterogeneity statistics, meaning there was not sufficient study power in this region to draw reliable conclusions. In comparison, the whole brain subgroup showed a statistically significant and large treatment effect (SMD = −1.04, 95% CI: −1.56 to −0.52; *p* < 0.0001), with moderate heterogeneity (Tau^2^ = 0.13; I^2^ = 24%), and Chi^2^ = 9.18 (df = 7; *p* = 0.24), showing some difference between studies. The subgroup differences test was statistically significant (Chi^2^ = 14.29; df = 6; *p* = 0.03; I^2^ = 58%), indicating that the treatment effect differed significantly based on the brain region under investigation. Although heterogeneity was low overall, these subgroup findings indicate that the brain region can potentially be a moderator of treatment effect, with certain regions having more and less robust protective effects than others (Figure 4). The 95% prediction interval ranges from −0.969 to −0.031 (with a standard deviation of 0.229), indicating that in similar future studies, the true effect size is likely to remain negative, although it may range from very small to large in magnitude. This suggests that intervention generally has a consistent beneficial effect.

### 3.10. Risk of Publication Bias

An examination of the studentized residuals in escape latencies of MWM analysis revealed that one study, Abdel-Zaher et al., 2017 [62], had a value larger than ±3.1340 and may be a potential outlier. Studentized residuals with cutoffs based on the t-distribution are used to identify potential outliers in meta-analysis. According to the Cook’s distances, one study (Abdel-Zaher et al., 2017 [62], where Citicoline as a pharmacological agent was used) could be considered to be overly influential. Egger’s regression test (intercept = 1.120, *p* = 0.273) indicated no significant funnel plot asymmetry, suggesting no strong evidence of publication bias. An examination of the studentized residuals in SOD analysis revealed that one study, Allagui et al., 2014 [63] (where melatonin as a pharmacological agent was used), had a value larger than ± 3.1440 and may be a potential outlier. According to the Cook’s distances, several studies, i.e., Bhargava et al., 2023 (*Cassia tora* extract) [14], Allagui et al., 2014 [63], Campos et al., 2022 (Chrysin) [68], and Prabhakar, 2020 (naringin) [87], could be considered to be overly influential. Egger’s regression test (intercept = −1.495, *p* = 0.146) indicated no statistically significant funnel plot asymmetry, suggesting no clear evidence of publication bias. CAT analysis revealed that none of the studies had a value larger than ± 3.1440, and hence there was no indication of outliers in the context of this analysis. According to the Cook’s distances, one study, Allagui et al., 2014 [63] (in which melatonin as a pharmacological agent was used), could be considered overly influential. Egger’s regression test (intercept = −0.610, *p* = 0.547) showed no significant funnel plot asymmetry, indicating no evidence of publication bias (Table 7). Funnel plots assessing publication bias (Figures S1–S3) are presented in the supplementary data.

## 4. Discussion

### 4.1. Evidence Summary and Interpretation

Aluminum, a widely distributed environmental element, has been implicated in neurotoxic outcomes [19], including, in particular, its impairment of cognitive functions, such as learning and memory [31]. Certain cognitive domains, including memory, working memory, and processing speed, are severely impaired by aluminum exposure [118]. It exerts neurotoxic effects by inducing oxidative stress, neuroinflammation, and neuronal degeneration and disrupting neurotransmitter metabolism and cytoskeleton integrity [19]. Our systematic review and meta-analysis consolidate the current pharmacological strategies to mitigate aluminum-induced neurotoxicity, offering a comprehensive view of their therapeutic potential.

The findings of our study reveal that several pharmacological agents are capable of significantly improving cognitive performance, as indicated by decreased escape latency in MWM and enhanced levels of anti-oxidant enzymes, like SOD and CAT. These improvements suggest that combating oxidative stress [85] is a central mechanism through which these treatments exert their neuroprotective effects. Agents like Memantine, *Hypericum perforatum* extract, and *Benincasa hispida* extract demonstrated strong efficacy in behavioral outcomes. Memantine is a moderate-affinity, uncompetitive N-methyl-D-aspartate receptor antagonist [119,120]. It improves learning and memory in aluminum-induced neurotoxicity [121,122,123,124] and reduces oxidative damage [125] in rodent models. Patients with moderate to severe AD-type dementia are commonly treated with Memantine [126]. It improves cognition in both AD and vascular dementia [126,127]. *Hypericum perforatum,* also known as St. John’s wort, is a well-known and widely used medicinal plant [128], and it improves learning and memory in aluminum-induced neurotoxicity models [69,129] and reduces oxidative stress [130,131]. *Benincasa hispida* is a well-known crop, and it is cultivated mostly for its fruits, but it is also known for its nutritional and medicinal qualities [132]. Fewer studies have evaluated the potential of *Benincasa hispida* to enhance memory and its protective effects against cognitive impairment and memory loss by modulating oxidative stress in D-galactose-induced aging in rats, suggesting a possible mechanism underlying its memory improvement properties [133]. Meanwhile, compounds like Chrysin and *Caesalpinia crista* extract exhibited prominent effects in biochemical assays by upregulating anti-oxidant defenses [20]. Chrysin is a promising phytochemical present in various plant sources, and it has been used extensively to treat a variety of degenerative diseases and brain disorders, like AD, PD, depression, anxiety, epilepsy, multiple sclerosis, traumatic brain and spinal cord injury, ischemic stroke, and brain tumors [134,135]. It has anti-inflammatory, cytotoxic, anti-oxidant, and disease-preventive properties [134]. It improves memory [136,137,138,139,140] in various neurotoxicity models. In aged mice brains, Chrysin improved memory in MWM and significantly improved SOD, CAT, and GPx activities [141]. *Caesalpinia crista* has anti-oxidant potential [142], and its ethanolic extract improved learning and memory performance in scopolamine-induced impairment by inhibiting brain AChE activity [143].

The meta-analysis of MWM escape latency (Figure 4) showed a significant treatment effect (SMD = 0.97, 95% CI: 0.74 to 1.19; *p* < 0.00001), demonstrating enhanced spatial memory and learning in aluminum-exposed rodents after intervention. This result was applicable across species, as both rats and mice demonstrated comparable improvements. While the transgenic mouse effect was not statistically significant, presumably because of sparse data, overall, pooled evidence strongly reinforces cognitive advantages to treatment. The SOD activity analysis (Figure 3), a key anti-oxidant enzyme, also supported treatment effectiveness. Significant improvements were noted especially in the hippocampus (SMD = −0.72; *p* = 0.0002) and the entire brain (SMD = −0.96; *p* = 0.02), indicating recovery of anti-oxidant defense in regions most susceptible to oxidative stress. Non-significant effects were noted in other brain regions, although this could be related to the regional heterogeneity of susceptibility or the small number of studies included. For CAT activity (Figure 4), an overall significant effect was noted again (SMD = −0.50; *p* < 0.0001), with the hippocampus and the whole brain showing the greatest effect, providing further support of the treatment reversing oxidative damage at the enzymatic level. The significant subgroup difference (Chi^2^ = 14.29; *p* = 0.03) indicates a region of the brain-dependent change in treatment efficacy. The hippocampus, known for its role in memory and its high oxidative vulnerability, appeared particularly responsive to treatment across both SOD and CAT outcomes. In the forest plot of the MWM analysis, the best results were observed for Memantine (6.4% weight) [38], *Hypericum perforatum* extract (5.8% weight) [69], and *Bennincasa hespidia* (5.4% weight) [90]. In the SOD analysis, the best results were observed for Chrysin (4.7% weight) in the cortex and (4.4% weight) in the hippocampus [68]. Similarly, in the CAT analysis, Chrysin (4.6% weight) [68] showed better results, followed by *Caesalpinia crista* methanolic extract (4.5% weight) in the hippocampus and the cortex, as well. It was observed that the number of animals used in the studies [38,69,89] was 24, 18, and 16, respectively, which are greater amounts than those reported in other studies. It is possible that the larger numbers result in a greater effect size in these studies. Moreover, SOD and CAT [68] showed enhanced results in eight animals, which is in range with other reported studies, so we can say that Chrysin improves learning and memory by increasing the anti-oxidant enzymes. Increased CAT activity is shown in [20], both in the hippocampus and in the cortex, and it also showed improvement in MWM (3.4% weight), with eight animals in each group. Considering the quality score of these studies, as mentioned in Table 5 [20,38,68,69,89], their scores were 5, 7, 7, 7, and 7 out of 8 points, and in Table 6, they received 10, 15, 13, 14, and 11 points out of 15, respectively. These findings suggest several possible treatment options for learning and memory impairments against aluminum-induced neurotoxicity. It is important to note that not all treatments yielded positive outcomes. For example, insulin and melatonin [60,84] did not significantly improve cognitive parameters in their respective studies. The differing results of melatonin in the two studies [60,63] may be attributed to variations in the animal models used, differences in routes of administration and neurotoxic agents used (AlCl_3_ and aluminum lactate), and differences in cognitive assessment responsiveness, all of which can influence its bioavailability, efficacy, and observed outcomes. While several studies were identified as potential outliers or overly influential based on studentized residuals and Cook’s distance, Egger’s regression results did not support the presence of significant publication bias.

Variation among pharmacological agents highlights the intricate nature of aluminum neurotoxicity and implies that different anti-oxidant or neuroprotective agents have varying degrees of efficacy in preventing aluminum-induced cognitive impairments. The impact of sample size, treatment duration, and methodological quality can be a factor. Studies with larger animal cohorts tended to report more substantial effects, most likely due to higher statistical strength. Comprehensive meta-analyses were challenging to conduct due to the variation in study designs, treatment procedures, and outcome reporting.

### 4.2. Methodological Considerations

In this review, we followed the CAMARADES checklist to evaluate the quality of studies [46,47]. The methodological quality of the included studies was generally higher; most of the studies showed strong adherence to quality criteria, with minor variation. The methodological quality score was a mean of 11.3 (Table 6). The systematic review showed the results for biochemical analysis, genetic expression analysis, behavioral analysis, and histological analysis of all of the included studies with their quality score. The point of concern is that although almost all studies had a statement that animals were randomly allocated, none mentioned the detailed criteria of random allocation. To reduce bias, randomization is the critical step; however, it is often conducted improperly and not reported thoroughly. According to the ARRIVE guidelines [144], blinding should be mentioned in detail within the studies to reduce bias. It is essential to ensure that the sample size is adequate for that purpose. Calculations are important to determine statistical significance and avoid ethical concerns [145]. A small sample size will lead to the risk of missing the accurate effect, and a large sample size will cause ethical concerns according to the ARRIVE guidelines [144]. The method for choosing the sample size must be mentioned in detail, but, unfortunately, none of the included studies described the process for determining the sample size. These omissions pose risks for bias and limit the strength of the conclusions that can be drawn.

Another particular area of concern was the use of anaesthetic agents. Among the selected studies, 25 did not specify the anaesthetic agent, 2 studies conducted only behavioral investigations without dissection of animals [38,61], 3 studies used ketamine in combination with xylazine [25,37,45], and 1 study used ketamine chloride [77]. Ketamine can induce neurotoxicity and lead to long-term neurofunctional impairment; meanwhile, it is also neuroprotective, as it reduces inflammation during painful stimuli [146]. The combination of ketamine and xylazine is effective for sedation, which is why proper dosage adjustments are necessary to mitigate these risks [147]. In three studies, ether was administered [59,65,89], which provides a quick onset of anesthesia. Sodium pentobarbital as an anaesthetic was used in three studies [69,90,95]. It is a widely used anesthetic in animal models, but it should be used carefully, as it can cause respiratory distress and deep sleep. Thiopental sodium was used in two studies [37,83]. Barbiturates like thiopental, pentobarbital, and phenobarbital may have neuroprotective effects [148]. In four studies, chloral hydrate [73,86,88,92] was utilized, which may be carcinogenic to rodents, which is an ethical concern. Furthermore, it can also result in morphological changes in hippocampal neurons, especially in aged rats, resulting in neuronal loss and microglia activation [149]. Halothane was used in one study [14] for its anaesthetic properties, but it also has neuroprotective properties [150]. The neuroprotective effects of anaesthetic agents, including barbiturates, propofol, xenon, and the majority of volatile anesthetics, like halothane, desflurane, sevoflurane, and isoflurane, protect cerebral tissue from damages like inflammation, apoptosis, degeneration, and energy failure, which can be brought on by chronic neurodegenerative diseases, stroke, ischemia, or trauma to the nervous system [151]. Urethane was utilized by one study [23], which is noted for having minimal changes in respiration and circulation, with physiological relevance in the data collection process [152]. Variability in euthanasia protocols introduces another layer of potential confounding. Future preclinical studies should prioritize using standardized, ethically sound anesthesia practices and transparently report these procedures.

### 4.3. Implications

The preclinical systematic review provides an ethical approach to synthesizing preclinical evidence by increasing sample size without increasing resources. This systematic review provides evidence for possible treatments for aluminum-induced neurotoxicity. The meta-analysis results demonstrated that these pharmacological agents can potentially reduce the risk of memory impairments in aluminum-induced neurotoxicity. Animal models play a crucial role in research on different human diseases, as they can reproduce the pathophysiology of human diseases [153]. Aluminum neurotoxicity is caused by the induction of aluminum chloride, aluminum gluconate [75,86], and aluminum lactate in selected studies. The mode of aluminum administration, like AlCl_3_ or aluminum lactate, and the variability in dosing regimens across studies introduce further heterogeneity. Aluminum exposure is linked to neurotoxicity and memory impairments, as it is involved in neuroinflammation and structural changes in the brain, which induce cognitive dysfunction. Treatment requires pharmacological agents that can cross the BBB [154]. It works as a barrier to protect the brain from harmful substances while preventing certain drugs from entering the brain, reducing their pharmacological effect. Inadequate drug delivery into the brain results in reduced therapeutic efficacy and side effects due to a buildup in other organs and tissues [155]. Although this systematic review provides a list of therapeutic agents that can improve learning and memory, the mechanism through which most cross the BBB is still unknown. Further studies should be performed to evaluate the active ingredients that cross the BBB and focus on the possibilities of maximizing brain bioavailability. Additionally, our findings suggest the possibility that younger animals are more susceptible to aluminum-induced cognitive impairments. This is important when translating findings to human populations, particularly regarding pediatric exposure. This study will help researchers further study the identified treatments in the development of drugs and give a future direction to research aluminum-induced neurotoxicity.

## 5. Conclusions

A total of 45 studies investigating pharmacological interventions for aluminum-induced neurotoxicity were reviewed in this study. Despite the variability across studies, the methodological quality was adequate, and using a random effects model helped account for the observed heterogeneity. According to this study, aluminum exposure impairs learning and memory, most likely because of oxidative stress, neuroinflammation, and apoptosis, while several pharmacological agents can mitigate these effects. The therapeutic results were significantly influenced by improvements in anti-oxidant defenses, primarily through the improvement of enzymes like SOD and CAT. Differences in treatment efficacy appeared to be influenced by factors like animal model characteristics, drug formulation, dosing regimen, and study duration. Out of a total of 39 pharmacological agents investigated, most agents significantly improved learning and memory, except for insulin and melatonin. Memantine, *Hypericum perforatum* extract, and *Bennincasa hespidia* showed the most prominent improvement in memory, while Chrysin showed notable efficacy in restoring levels of anti-oxidant enzymes. This meta-analysis presents strong evidence that treatment has a significant effect on spatial memory performance and anti-oxidant enzyme activity in aluminum-exposed models. The most significant effects were noted in the hippocampus and the whole brain, highlighting their pivotal position in both cognitive impairment and oxidative stress. These results support the promise of selective therapeutic approaches to preventing aluminum-induced neurotoxicity through behavioral and biochemical restoration. Overall, Egger’s regression analyses suggest the absence of publication bias across the included studies, despite some influential data points.

Although this review highlights promising therapeutic options, gaps remain in our understanding, particularly regarding the ability of these agents to cross the BBB and their exact molecular mechanisms of action, except for approved drugs that are repurposed with known safety and BBB permeability profiles. Future research should prioritize identifying active compounds capable of improving brain bioavailability and elucidating the signaling pathways involved in aluminum-induced cognitive decline. This study provides a valuable framework for guiding future drug development to prevent or treat learning and memory deficits caused by aluminum neurotoxicity.

## Figures and Tables

**Table 1:** Research question formulation and criteria for inclusion and exclusion of studies, following PICO parameters.

Parameter	Inclusion Criteria	Exclusion Criteria
**Population**	Animal studies	Ex vivo and in vitro studies
**Intervention**	Aluminum exposure for ≥2 months, and the treatment agent used	Aluminum exposure for <2 months, aluminum exposure combined with any other neurotoxin
**Comparison**	Aluminum group, along with the treatment group	No treatment in the aluminum group
**Outcomes**	Assessment of learning and memory	No learning and memory assessment

**Table 7:** Risk of publication bias.

Parameter	Number of Studies	Egger’s Test (*p* Value)
MWM escape latencies	28	1.120 (0.273)
SOD levels	21	−1.495 (0.146)
CAT levels	18	−0.610 (0.547)

## Supplementary Materials

The following supporting information can be downloaded at: https://www.mdpi.com/article/10.3390/brainsci15080849/s1, Figure S1: Funnel plot to assess publication bias in the Escape latencies of MWM outcomes; Figure S2: Funnel plot to assess publication bias in the SOD level outcomes; Figure S3: Funnel plot to assess publication bias in the CAT level outcomes.
