# Supplementary material for: A Systematic Review of Preclinical Studies Investigating the Effects of Pharmacological Agents on Learning and Memory in Prolonged Aluminum-Exposure-Induced Neurotoxicity
Source: Brain Sci. 2025 Aug 8;15(8):849. doi: 10.3390/brainsci15080849 (PMC12384838; doi:10.3390/brainsci15080849)
Supplement: Supplementary file 1 [file brainsci-15-00849-s001.zip › brainsci-3761777-supplementary.pdf]

A Systematic Review of Preclinical Studies Investigating the Effects of Pharmacological Agents on Learning and Memory in Prolonged Aluminum Exposure Induced Neurotoxicity

Supplementary Data

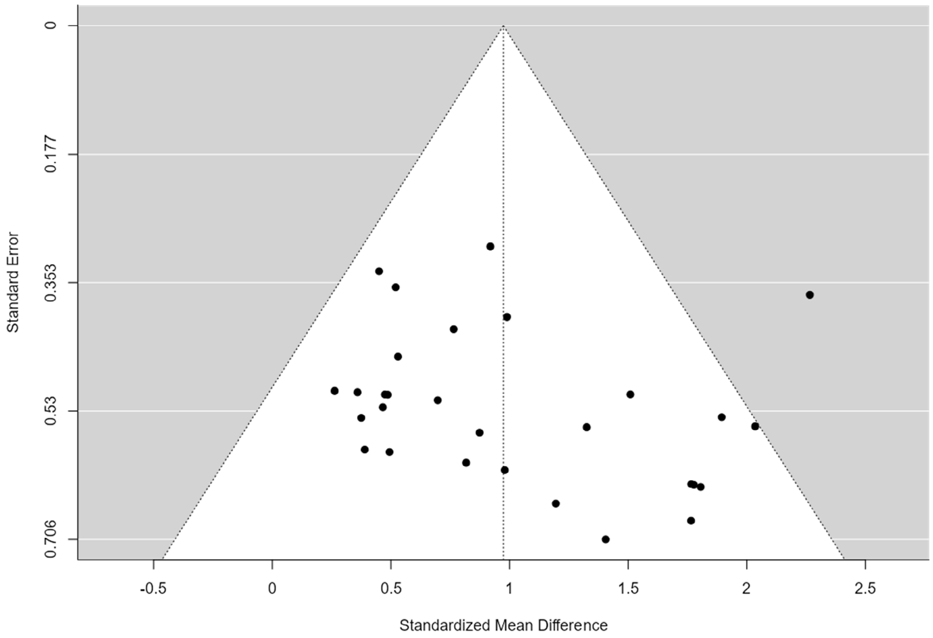

Figure S1 Funnel plot to assess publication bias in the Escape latencies of MWM outcomes

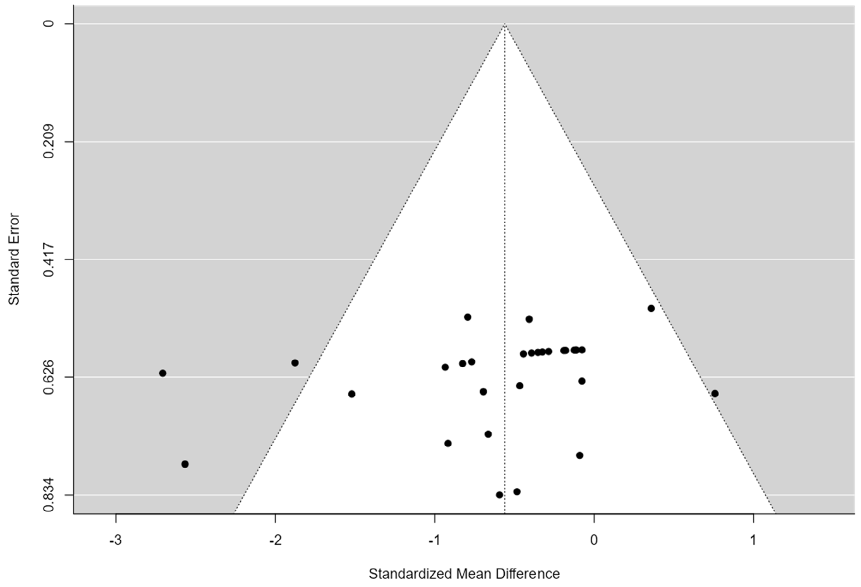

Figure S2 Funnel plot to assess publication bias in the SOD level outcomes

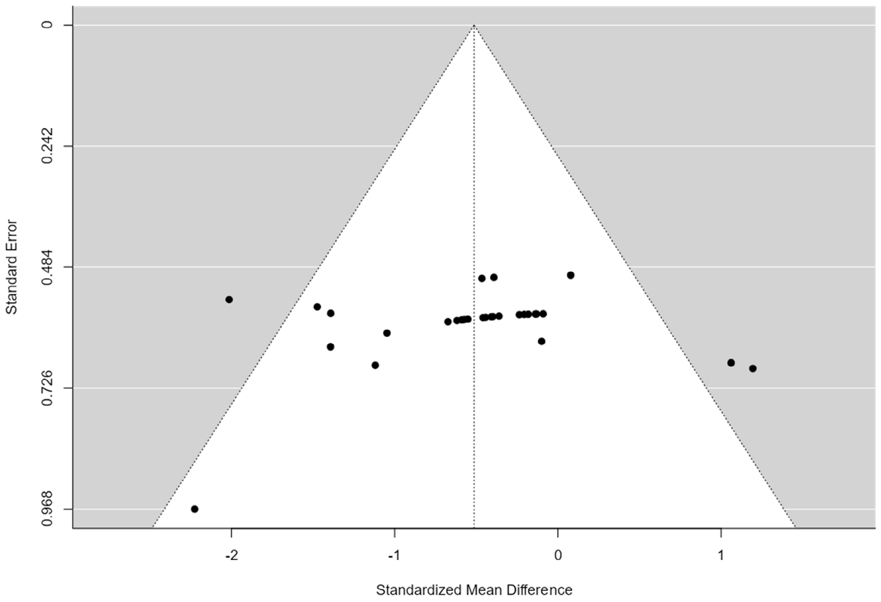

Figure S3 Funnel plot to assess publication bias in the CAT level outcomes
